# Metabolomic Signature of Early Vascular Aging (EVA) in Hypertension

**DOI:** 10.3389/fmolb.2020.00012

**Published:** 2020-02-07

**Authors:** Katarzyna Polonis, Renata Wawrzyniak, Emilia Daghir-Wojtkowiak, Anna Szyndler, Marzena Chrostowska, Olle Melander, Michał Hoffmann, Marta Kordalewska, Joanna Raczak-Gutknecht, Ewa Bartosińska, Roman Kaliszan, Krzysztof Narkiewicz, Michał J. Markuszewski

**Affiliations:** ^1^Department of Hypertension and Diabetology, Medical University of Gdansk, Gdansk, Poland; ^2^Department of Laboratory Medicine and Pathology, Mayo Clinic, Rochester, MN, United States; ^3^Department of Biopharmaceutics and Pharmacodynamics, Medical University of Gdansk, Gdansk, Poland; ^4^Department of Clinical Sciences, Lund University, Malmö, Sweden

**Keywords:** early vascular aging, metabolomics, arterial stiffness, pulse wave velocity, phospholipid metabolism

## Abstract

Arterial stiffening is a hallmark of early vascular aging (EVA) syndrome and an independent predictor of cardiovascular morbidity and mortality. In this case-control study we sought to identify plasma metabolites associated with EVA syndrome in the setting of hypertension. An untargeted metabolomic approach was used to identify plasma metabolites in an age-, BMI-, and sex-matched groups of EVA (*n* = 79) and non-EVA (*n* = 73) individuals with hypertension. After raw data processing and filtration, 497 putative compounds were characterized, out of which 4 were identified as lysophosphaditylcholines (LPCs) [LPC (18:2), LPC (16:0), LPC (18:0), and LPC (18:1)]. A main finding of this study shows that identified LPCs were independently associated with EVA status. Although LPCs have been shown previously to be positively associated with inflammation and atherosclerosis, we observed that hypertensive individuals characterized by 4 down-regulated LPCs had 3.8 times higher risk of EVA compared to those with higher LPC levels (OR = 3.8, 95% CI 1.7–8.5, *P* < 0.001). Our results provide new insights into a metabolomic phenotype of vascular aging and warrants further investigation of negative association of LPCs with EVA status. This study suggests that LPCs are potential candidates to be considered for further evaluation and validation as predictors of EVA in patients with hypertension.

## Introduction

Arterial stiffness, which is described by structural and functional properties of arteries, is a biomarker of vascular aging that reflects both chronologic (metric) and biological aging (Nilsson et al., 2009). Carotid-femoral pulse wave velocity (cfPWV) is a widely accepted gold standard for assessing arterial stiffness in clinical practice (Van Bortel et al., 2012). Increased pulse wave velocity (PWV) is a commonly recognized independent and strong predictor for cardiovascular morbidity and mortality, both in the general population and in individuals with hypertension (Willum-Hansen et al., 2006; Vlachopoulos et al., 2010). According to the European Society of Hypertension (ESH) guidelines, cfPWV values >10 m/s are an indication of subclinical organ damage (Williams et al., 2018); however the interpretation of cfPWV values should always be done with caution as it does not take into account an individual's age and blood pressure category.

High blood pressure is a major risk factor for cardiovascular disease, and therefore hypertensive populations may be considered models of accelerated vascular aging (Schiffrin, 2004; Kaess et al., 2012; Safar et al., 2018). Indeed, growing evidence shows that reducing blood pressure is associated with decreased arterial stiffness as measured by PWV and improved overall survival in patients either treated or untreated for other cardiovascular risk factors (Asmar, 2001; Dudenbostel and Glasser, 2012). Among many other cardiovascular risk factors, obesity (Sutton-Tyrrell et al., 2001; Safar et al., 2018), impaired glucose tolerance and diabetes (Stehouwer et al., 2008), dyslipidemia (Asmar, 2001; Wilkinson and Cockcroft, 2007), and smoking (Doonan et al., 2010; Dudenbostel and Glasser, 2012) are also linked with an accelerated process of vascular aging. In this context, arterial stiffness is thought to reflect a cumulative damage of risk factors on the arterial wall along with metric aging, and thus represent an intermediate and critical step leading to cardiovascular events (Nilsson et al., 2009).

These observations led to the concept of early vascular aging (EVA) syndrome, which is generally defined as a state of accelerated vascular aging that cannot be explained solely by metrical age (Nilsson, 2008b). EVA can be diagnosed in individuals who present with abnormally high arterial stiffness with respect to their age and sex category. In younger subjects, cfPWV values lower than 10 m/s may suggest accelerated vascular aging, but normal trajectory of vascular aging in older subjects (Laurent et al., 2019). Distinguishing between these two states is clinically critical as it dictates the most appreciate management and treatment. Therefore a concept of EVA is recently gaining more clinical recognition as a tool to efficiently identify hypertensive individuals at elevated risk of developing adverse cardiovascular outcomes (Nilsson et al., 2013; Antza et al., 2018; Laurent et al., 2019).

Conceivably, identifying biomarkers of EVA, ideally prior to a complete or in early clinical EVA manifestation, may potentially detect individuals at risk of arterial damage and prompt a clinical intervention to address cardiovascular risk factors. This in consequence may deaccelerate a trajectory to EVA and thus reduce a risk of CVD. The mechanisms underlying a development of EVA in hypertensive subjects, however, still remain unclear and likely to involve complex, multilevel interactions between blood pressure and other cardiovascular risk factors.

Metabolomics is a high-throughput mass spectrometry method that offers a powerful platform tools to explore metabolic alterations associated with pathophysiological states. Several studies have indeed proven that metabolomics provide important insight into cardiovascular disease pathogenesis such as cardiac hypertrophy, heart failure, coronary heart disease, cardiometabolic disease, and hypertension as well as identify new potential cardiovascular disease biomarkers (Nikolic et al., 2014; Tzoulaki et al., 2018). Metabolomic profiling is also thought to integrate valuable information reflecting genomic, epigenetic, and transcriptomic variation with the effect of environmental exposures such as diet, physical activity which is unique for each individual (McGarrah et al., 2018; Leon-Mimila et al., 2019). Therefore, metabolomics is a powerful tool to accelerate characterization and providing insights into multiple aspects of complex phenotypes such as cardiovascular physiology.

In this case-control study we sought to explore metabolomic signatures of EVA syndrome in hypertension and identify metabolites that may potentially serve as prognostic and/or early diagnostic markers of EVA status. Detection of unfavorable LPC profiles prior to or at early stage of EVA manifestation would allow taking appropriate clinical care measures to address an individual's risk of cardiovascular disease. Our study enriches the metabolomic concept of vascular aging and provides a framework for further research. We also anticipate that the metabolomic approach will lead to identification of novel molecular targets for therapeutic options aimed at slowing the arterial aging process and designing personalized cardiovascular protection approaches.

## Materials and Methods

### Study Population

This case-control study included 152 individuals derived from the CARE NORTH cohort for whom carotid-femoral pulse wave velocity (cfPWV) parameter, a measure of arterial stiffness, was available. Cases were selected on the basis of having the outcome (early vascular aging, EVA status) and compared with a control group matched primarily for age, BMI, and sex. The EVA status of each patient was determined at the baseline of the CARE NORTH study as described in detail in Early Vascular Aging (EVA) Syndrome Definition.

The CARE NORTH study is a prospective cohort of 854 hypertensive subjects recruited between 2009 and 2011 at the Medical University of Gdansk, Poland (Polonis et al., 2018; Swierblewska et al., 2018). All participants completed baseline examinations including a medical history, physical examination, and had plasma samples collected. The study was conducted in accordance with the Declaration of Helsinki and approved by the Independent Committee of Bioethical Research at the Medical University of Gdansk (NKEBN/285/2009). All subjects gave written informed consent in accordance with the Declaration of Helsinki.

### Early Vascular Aging (EVA) Syndrome Definition

EVA syndrome was diagnosed in patients with cfPWV values >2 standard deviations (SD) above the mean reference values according to an age and blood pressure category, as described in the Reference Values for Arterial Stiffness Collaboration (Mattace-Raso et al., 2010). Briefly, reference values were defined based on the cfPWV distribution observed in the population of 11,092 individuals of both sexes and presenting CVD risk factors that have been shown to have no independent effect on cfPW (Mattace-Raso et al., 2010). In this study, a blood pressure category was assigned based on the average brachial blood pressure (office BP) measured after 15 min of resting in a supine position prior to an arterial stiffness examination. The cfPWV measurements were performed using a SphygmoCor® device (Atcor, Sydney, Australia).

### Plasma Samples Preparation and Analytical Measurements

EDTA-plasma samples were collected after an overnight fasting and stored at −80°C for up to 5 years prior to metabolomic analysis. Plasma samples were thawed on ice for 2 h before a metabolite extraction procedure. Plasma samples (50 μl) were deproteinizated with 150 μl of acetonitrile, vortexed for 5 min and kept at −20°C for 1 h. Next, samples were centrifuged (13,000 × g, 15 min, 4°C) and obtained supernatants were filtered (4 mm nylon syringe filters, 2 μm, Thermo Scientific, USA). Plasma extracts (2 μL) were analyzed using LC-TOF/MS technique.

To monitor the analytical systems and methods stability during the analyses, quality control (QC) samples were prepared by mixing equal volume (5 μL) of each plasma sample. The QC samples were prepared with the same procedure as other plasma samples and analyzed after every 10 plasma samples in the sequence run.

Plasma metabolomic analyses were performed with Agilent 1200 Series LC system (Agilent Technologies, Waldbronn, Germany) coupled with Agilent 6224 Series TOF analyzer (Agilent Technologies, Waldbronn, Germany) and equipped with a dual electrospray ionization source (dual ESI). All plasma samples were randomly analyzed in two separated sequences (the first in positive and the second in negative ion mode). The chromatographic separation was performed at the temperature of 50°C using Zorbax Extend Rapid Resolution HT C18 column (10 cm × 2.1 mm, 1.8 μm, Agilent Technologies, Waldbronn, Germany). As a mobile phase, 0.1% formic acid (97%, Alfa Aesar, Germany) in deionized water (A) and 0.1% formic acid in acetonitrile (B) were used. A flow rate was set at 0.35 mL/min. The gradient elution was carried out from 5 to 100% of B in 8.0 min, and then kept for 5 min at 100% of B. The column was re-equilibrated for 7 min.

Plasma extracts were analyzed with the use of scan mode in the range of 50–1,100 *m/z* (mass to charge ratio) in positive and negative ionization modes, separately. The scan rate was set at 1.0 spectra/second. To ensure accurate mass measurements, four reference masses (121.0509 and 922.0098 *m/z* in the positive mode, and 112.9856 and 1033.9881 *m/z* in the negative ionization mode) were automatically delivered using dual ESI source during sample analyses. Capillary voltage, fragmentor, nebulizer gas flow rate, and pressure were set to 3,250 V, 150 V, 11 L/min, and 50 psig, respectively.

### Data Processing and Metabolite Identification

Raw datasets were processed with the use of Molecular Feature Extraction (MFE) algorithm in MassHunter Qualitative Analysis B.06.00 software (Agilent Technologies, Waldbronn, Germany) in order to conduct background clean-up and extract all signals measured in plasma samples. The MFE parameters including a noise threshold, possible adducts, and an isotopic distribution was similar to previously described (Ciborowski et al., 2012). After MFE data extraction, each potential compound was described by monoisotopic mass, retention time, and abundance. Afterwards, alignment procedure was applied with the use of Mass Profiler Professional B.02.01 (Agilent Technologies, Waldbronn, Germany). The applied parameters for retention time and mass correction were set to 1% and 5 ppm, respectively. The alignment step provides the opportunity to address a retention time and measured mass shift during LC–MS analyses, and ensures that each detected signal is denoted as the same potential compound in all plasma samples.

The next step of the data treatment procedure constituted filtration according to the recommended quality assurance (QA) criteria including both frequency (at least 50%) and coefficient of variation (CV) value (<20%) in QC samples (Dunn et al., 2011). The second filtration step was applied to keep only the features present in 80% of samples in at least one of the compared groups (i.e., in 80% of samples in EVA or non-EVA group). The normalization procedure was performed using MS Group Useful Signal (MSGUS) approach (Warrack et al., 2009).

Analytical signals that passed data alignment and filtration criteria were putatively characterized based on monoisotopic mass, formula, isotopic distribution, and hits found in publicly available databases, such as: METLIN (http://metlin.scripps.edu), HMBD (http://hmdb.ca), PubChem (http://pubchem.ncbi.nlm.nih.gov/), KEGG (http://genome.jp/kegg), Lipid MAPS (http://www.lipidmaps.org) with the use of CEU mass mediator tool version 2.0 (Gil de la Fuente et al., 2018) (http://ceumass.eps.uspceu.es/mediator/) (Supplemental Material 2). The identity of metabolites which clearly differentiated EVA and non-EVA patients was confirmed by LC-MS/MS consisted of an Agilent 1260 Series LC system (Agilent Technologies, Waldbronn, Germany) and QTOF (model 6546, Agilent Technologies, Waldbronn, Germany). Analytical measurements were repeated with identical chromatographic parameters as in the primary untargeted analyses. The selected ions were targeted for collision-induced dissociation (CID) fragmentation based on the previously determined accurate mass and retention time. Comparison of the structure of the proposed metabolite with the fragments obtained during MS/MS analyses can confirm the identity.

### Statistical Analysis

Principal component analysis (PCA) was used to evaluate quality of analyses and general trends in the data. Hotelling's T2 range was used to detect potential outliers. Least Absolute Shrinkage and Selection Operator (LASSO) were used to select metabolites which contribute the most to recognition between non-EVA and EVA group. A reproducibility of the results was assessed with a resampled-based bootstrap procedure (Pineda et al., 2014).

LASSO is a regularization-based technique allowing variables selection together with a model development. C y response as a dependent variable, the penalty term (1) is added to the log-likelihood function used in classical logistic regression (2) to form LASSO penalized logistic regression (3),

(1) $$\begin{array}{l} \overset{P}{\underset{j=1}{\sum }}|\beta_{j}|\leq \lambda \end{array}$$

(2) $$\begin{array}{l} \ln\;L\left(y_{i},\beta \right)=\overset{n}{\underset{i=1}{\sum }}\left[y_{i}\ln\;\left(\frac{\pi }{1-\pi_{i}}\right)\right]+\overset{n}{\underset{i=1}{\sum }}\ln\;\left(1-\pi_{i}\right) \end{array}$$

(3) $$\begin{array}{l} g\left(y_{i};\beta ;\lambda \right)=L\left(y_{i},\beta \right)+\lambda \overset{P}{\underset{j=1}{\sum }}|\beta_{j}| \end{array}$$

where β represents regression coefficients, y_*i*_ is the response variable for *i*th individual, *p* denotes a predictor variable, *n* refers to sample size and λ is a penalty term (also known as a tuning parameter) (Pineda et al., 2014).

The λ penalty term controls the amount of shrinkage imposed on model's regression coefficients according to Equation (1). If λ is large, the coefficients are penalized highly toward zero (all absolute coefficients are penalized), whereas low value of λ imposes little penalty on the coefficients. The most common technique to estimate λ is cross-validation (however other criteria also exist e.g., AIC, *Akaike Information Criterion*; BIC, *Bayesian Information Criterion*). Considering large sample space (high-dimensionality), the advantage of LASSO lies in the development of more stable models via reduction of the variance at the cost of introducing bias into model's parameters. Since it is useless to calculate standard errors for biased parameters, we used a resampled-based bootstrap procedure to assess the reproducibility of the results (Pineda et al., 2014; R Core Team, 2014). For each resample, the LASSO model, adjusted for age, sex, and BMI, was developed and the reproducibility of results was calculated as a proportion (per 1,000 times) when each metabolite was introduced into the LASSO model.

All analyses were performed with “penalized” and “rcorr” package in R Core Team (2014) to fit the LASSO model and perform the correlation analysis, respectively. PCA modeling and plotting were performed with the use of SIMCA software (version P13, Umetrics, Umeå, Sweden).

An unsupervised hierarchical cluster analysis with Ward's method for defining distances between clusters was used to determine a metabolomic signature of EVA. Before clustering the data was standardized to a mean of 0 and a standard deviation of 1 where the standard deviation and mean were computed for the raw data. Binary logistic regression was applied to calculate unadjusted and adjusted odds ratios (ORs), to assess the association between given clusters (profiles) and a risk of EVA.

## Results

### Characteristics of the Study Population

Out of 854 hypertensive individuals in the CARE NORTH cohort, 79 EVA and 73 non-EVA subjects were included into this study that aimed to explore a metabolomic signature associated with EVA syndrome. Non-EVA and EVA group were matched for age (44.0 ± 14.8 vs. 43.0 ± 13.5, *P* = 0.652), BMI (28.9 ± 4.0 vs. 28.6 ± 4.7, *P* = 0.662), and sex distribution (73 vs. 76% male, *P* = 0.637). There was no significant difference in the frequency of active smoking status, diabetes mellitus type 2 (DM2), cardiovascular disease (CVD), the usage of calcium channel blockers (CCB), beta blockers (BB), diuretics, hypolipidemic agents, and acetylsalicylic acid (ASA) between groups (*P* > 0.05); however, there were 11% more individuals receiving ACE-1/ARB treatment in the non-EVA compared to EVA group (*P* = 0.046). Non-EVA group was also characterized by significantly higher office systolic (SBP) and office diastolic (DBP) blood pressure measured prior to arterial stiffness examination. A detailed baseline study characteristic is presented in Table 1.

**Table 1:** Study population characteristics.

	**Non-EVA group** ***n* = 73**	**EVA group** ***n* = 79**	***P*-value**
Age (years)	44.0 ± 14.8	43.0 ± 13.5	0.652
BMI (kg/m^2^)	28.9 ± 4.0	28.6 ± 4.7	0.662
Male sex *n* (%)	53 (73%)	60 (76%)	0.637
Active smoking *n* (%)	11 (15%)	14 (18%)	0.634
cfPWV (m/s)	9.5 ± 2.3	11.3 ± 2.3	2.1 ×10^−6^
Office SBP (mm Hg)	134.9 ± 17.3	126.9 ± 11.9	0.001
Office DBP (mm Hg)	75.9 ± 11.8	70.7 ± 9.5	0.003
ACE-1/ARB *n* (%)	65 (89%)	60 (77%)	0.046
CCB *n* (%)	42 (58%)	37 (47%)	0.214
Beta blockers *n* (%)	42 (58%)	41 (53%)	0.540
Diuretics *n* (%)	45 (62%)	37 (47%)	0.067
Hypolipidemic *n* (%)	51 (70%)	50 (63%)	0.391
ASA *n* (%)	17 (23%)	16 (21%)	0.680
CVD *n* (%)	4 (5%)	5 (6%)	0.824
DM2 *n* (%)	15 (21%)	10 (13%)	0.189

*P-values calculated by t-test or chi-square test. Data presented as mean ± standard deviation or number (%) for each group. BMI, body mass index; cfPWV, carotid-femoral pulse wave velocity; office SBP/DBP, systolic/diastolic blood pressure measured in a supine position prior to arterial stiffness examination; ACE-1/ARB, angiotensin-converting-enzyme inhibitor/angiotensin II receptor blockers; CCB, calcium channel blocker; diuretics, thiazides and/or aldosterone antagonists; hypolipidemic treatment, statins and/or fibrates; ASA, acetyl salicylic acid treatment; DM2, diabetes mellitus type 2; CVD, cardiovascular disease defined as coronary heart disease and/or cerebrovascular disease*.

### Metabolites Identification and Their Inference on EVA and Non-EVA Status

**Figure 1:**
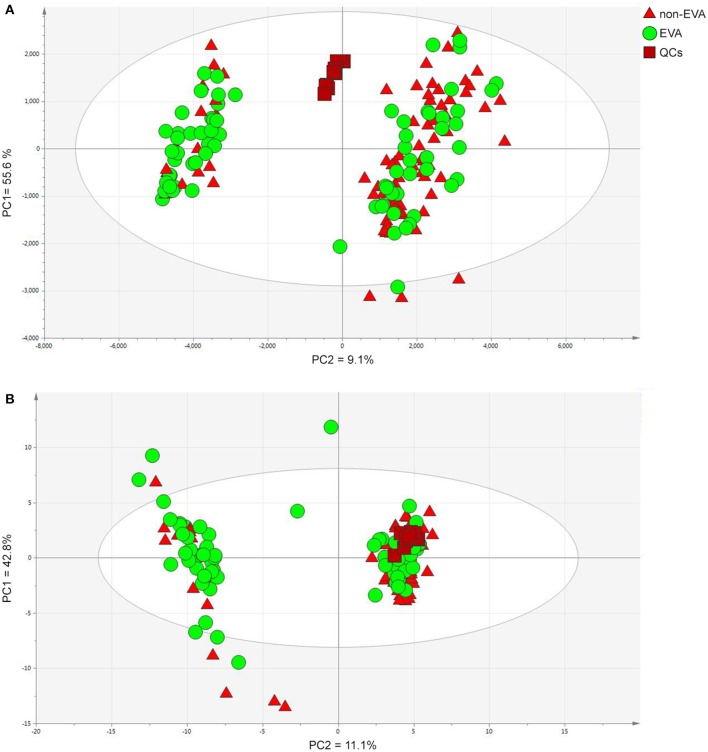
PCA models for dataset after filtration obtained in positive **(A)** and negative **(B)** ion modes. PC1/2—principal component 1/2. Data were log transformed and Pareto or UV scaled in the case of positive and negative ionization modes, respectively. **(A)** PCs contribution (PC1 = 55.6 % and PC2 = 9.1%), **(B)** PCs contribution (PC1 = 42.8%, PC2 = 11.1%).

As a result of the raw data processing, 497 measured compounds, including 374 in positive ion mode and 123 in negative ion mode, were putatively characterized. Principal component analysis (PCA) showed a correct clustering of the quality control (QC) samples, and thus confirmed low analytical variation. No extreme outliers were observed (Figure 1). Least Absolute Shrinkage and Selection Operator model (LASSO) identified 4 metabolites which clearly differentiated EVA and non-EVA individuals: lysophosphatidylcholine [LPC (18:2)], lysophosphaditylcholine [LPC (16:0)], lysophosphatidylcholine [LPC (18:0)], and lysophosphatidylcholine [LPC (18:1)]. The reproducibility of these 4 metabolites ranged between 39 and 65%. The detailed identification information of the selected metabolites is presented in Table 2.

**Table 2:** The detailed identification of metabolites contributing into the classification between non-EVA and EVA group.

**Name**	**Mass**	**RT**	**Formula**	**Formula score**	**LIPIDMAPS ID and InChiKey**	**Fragmentation pattern**	**MSI identification level**	**CV in QCs**	**Change EVA vs. non-EVA**	**Bootstrap-based reproducibility of metabolite**
LPC (18:0)	523.3646	8.5	C26H54NO7P	98%	LMGP01050026 IHNKQIMGVNPMTC-RUZDIDTESA-N	524.3710, 506.3606, 285.2799, 184.0733, 104.1069, 86.0962, 60.0805	2	4.3%	Down	58%
LPC (18:1)	521.3489	7.8	C26H52NO7P	97%	LMGP01050138 PZRFVAHZNWPPAC-VBKSFVIKSA-N	522.3556, 504.3450, 258.1102, 184.0735, 104.1070, 86.0963, 60.0805	2	4.8%	Down	39%
LPC (18:2)	519.3327	7.2	C26H50NO7P	98%	LMGP01050035 SPJFYYJXNPEZDW-FTJOPAKQSA-N	520.3402, 502.3296, 258.1103, 184.0736, 104.1071, 86.0964, 60.0805	2	3.8%	Down	52%
LPC (16:0)	495.3329	7.6	C24H50NO7P	99%	LMGP01050018 ASWBNKHCZGQVJV-HSZRJFAPSA-N	496.3403, 478.3296, 258.1102, 184.0736, 104.1070, 86.0963, 60.0804	2	4.6%	Down	65%

*LPC, lysophosphaditylcholines; RT, retention time; MSI, Metabolomics Standard Initiative; CV, coefficient of variation; QCs, quality control samples; EVA, early vascular aging*.

The individual fragmentation spectra of statistically significant metabolites (lysophosphatidylocholines-LPCs) have been submitted at https://mona.fiehnlab.ucdavis.edu/. The obtained accession numbers for LPC (16:0), LPC (18:2), LPC (18:1), LPC (18:0) were assigned as MoNA011502, MoNA011503, MoNA011504, MoNA011505, respectively. All the data generated and the list of putatively characterized metabolites in this study are available at MetaboLights database (www.ebi.ac.uk/metabolights/MTBLS1359) (Haug et al., 2013).

**Figure 2:**
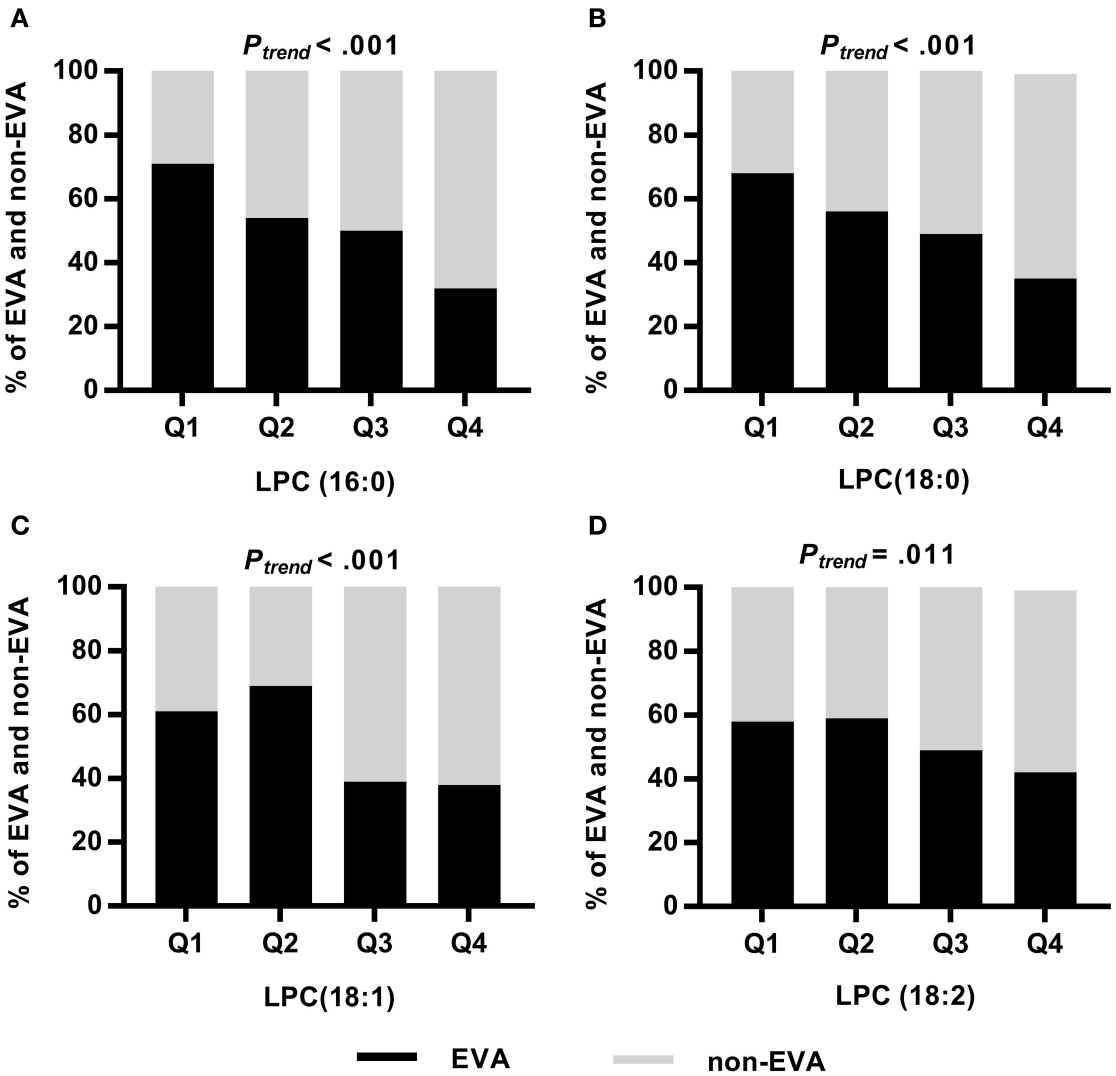
Frequency of EVA and non-EVA individuals across quartiles of a given LPC: LPC (16:0) **(A)**, LPC (18:0) **(B)**, LPC (18:1) **(C)**, and LPC (18:2) **(D)**. *P*-values were calculated by Chi square test for trend.

Analyzing the frequency of EVA status according to quartiles of LPCs, we found significant trends showing that lower levels of LPCs were associated with higher rates of EVA (Figure 2). In the first quartile of LPC (16:0), LPC (18:0), LPC (18:1), and LPC (18:2) there were 39, 32, 23, and 16% more EVA than non-EVA individuals, respectively, as compared to the fourth quartile. Levels of LPC (16:0), LPC (18:0) and LPC (18:1) were also positively correlated with cfPWV (*P* < 0.001 for each metabolite), office SBP (*P* < 0.001 for each metabolite), and office DBP (*P* < 0.001 for each metabolite) in the entire study group, regardless of the EVA status. LPC (18:2) was negatively correlated with cfPWV (*P* = 0.016) and showed no association with either office SBP or DBP (Supplementary Figure 1). Associations between metabolites levels and cfPWV in the EVA group were consistent with these observed for the entire group. None of LPCs were significantly correlated with office SBP, and only LPC (16:0) showed a significant correlation with office DBP in the EVA group (Supplementary Figure 2).

### Metabolomic Profiles Definition

To determine a metabolomic signature of EVA based on 4 selected LPCs, we performed an unsupervised hierarchical cluster analysis. A predefined number of clusters were 2, 3, and 4. Clusters including <10 subjects were excluded from further analysis. Clusters with the lowest percentage of EVA individuals were treated as a reference level to calculate ORs of EVA in comparison to other clusters.

Model 1 with 4 generated clusters that showed the highest prediction value of EVA status as determined by the highest numerical value of unadjusted and adjusted ORs (Table 3). Cluster 3 and cluster 4 of model 1 comprised 5 and 2 individuals, respectively, and thus were not included in any further analyses.

**Table 3:** Rates of EVA syndrome in clusters generated by an unsupervised hierarchical clustering, and unadjusted and adjusted ORs of EVA.

**Model 1 (4 clusters)**			**Model 2 (3 clusters)**			**Model 3 (2 clusters)**		
	***n***	**% EVA**		***n***	**% EVA**		***n***	**% EVA**
Cluster 1	107	61%	Cluster 1	107	61%	Cluster 1	107	61%
Cluster 2	38	29%	Cluster 2	43	33%	Cluster 2	45	31%
Cluster 3	5	60%	Cluster 3	2	0%			
Cluster 4	2	0%						
**Odd ratios (ORs): cluster 1 vs. cluster 2**								
OR_unadj_ 3.8 (1.7–8.5), *P* = 0.001			OR_unadj_ 3.2 (1.5–6.7), *P* = 0.002			OR_unadj_ 3.4 (1.6–7.2), *P* = 0.001		
OR_adj1_ 5.5 (2.1–14.4), *P* < 0.001			OR_adj1_ 4.4 (1.8–10.9), *P* = 0.001			OR_adj1_ 4.8 (1.9–11.7), *P* < 0.001		
OR_adj2_ 4.9 (1.7–13.8), *P* = 0.003			OR_adj2_ 3.9 (1.5–10.6), *P* = 0.007			OR_adj2_ 4.2 (1.6–11.4), *P* = 0.004		

*adj1, adjusted for age and sex; adj2, additionally adjusted for ACE-1/ARB, CCB, diuretics, hypolipidemic, and ASA treatment*.

**Figure 3:**
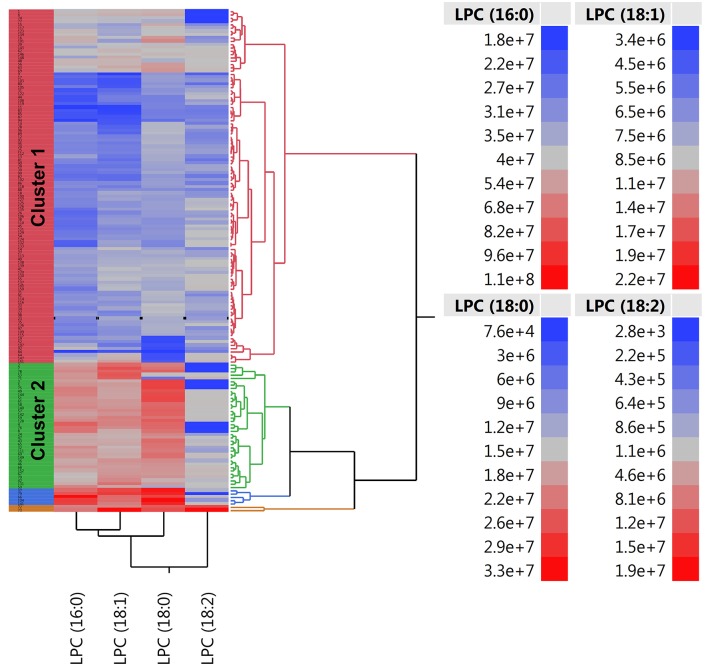
Two-way hierarchical clustering heatmap of plasma LPC (16:0), LPC (18:0), LPC (18:1), and LPC (18:2). Red and blue color represents high and low abundance of metabolites, respectively.

There were twice as many individuals with EVA in cluster 1 than in cluster 2 (61 vs. 29%, *P* < 0.001) and the levels of LPC (16:0), LPC (18:0), LPC (18:1), and LPC (18:2) were 50, 50, 40, and 10% lower, respectively (Figure 3). Individuals in cluster 1 were significantly younger (39.3 ± 13.2 years vs. 54.1 ± 11.1 years, *P* < 0.001), predominantly men (83 vs. 55%, *P* < 0.001), and had significantly lower systolic and diastolic blood pressure compared to those in cluster 2. Conversely, in cluster 2 there was a significantly higher rate of individuals receiving ACE-1/ARB, CCB, diuretics, ASA, and hypolipidemic treatment than in cluster 1 (Supplementary Table 1).

Individuals in cluster 1 had 3.8 times greater risk of EVA than individuals from cluster 2 (OR = 3.8, 95% CI 1.7–8.5, *P* < 0.001). Additional adjustment for age, sex, and pharmacological treatment provided numerically higher OR of 4.9 (95% CI 1.7–13.8, *P* = 0.003), meaning that individuals characterized by lower levels of LPCs were at 4.9 times higher risk of developing EVA (Table 3). We also observed a higher increase of cfPWV with each year in cluster 1 than in cluster 2 (β = 0.11 ± 0.02, *P* < 0.001 vs. β = 0.14 ± 0.01, *P* < 0.001), however the difference between the slopes was not statistically significant (*P* = 0.181).

## Discussion

In this study we used an untargeted metabolomic approach to identify circulating plasma metabolites associated with EVA syndrome in the setting of hypertension. We demonstrate that hypertensive individuals with metabolomic profiles characterized by 4 down-regulated plasma lysophosphatidylcholines (LPC 16:0, LPC 18:0, 18:1, and 18:2) were at greater risk of developing EVA syndrome. This finding suggests that these metabolites may be potential candidates for further evaluation and validation as early predictors of EVA in patients with hypertension. Furthermore, we anticipate that changes in blood lipid composition observed in EVA syndrome may facilitate identifying attractive targets in cardiovascular prevention strategies.

Vascular aging is a gradual process involving biochemical, enzymatic, and cellular alterations of the vasculature (Kotsis et al., 2011; Nilsson et al., 2013). Early (premature) vascular aging, namely EVA, may be characterized by increased arterial stiffness, endothelial dysfunction, impaired vasodilatation, chronic inflammation, and dyslipidemia (Nilsson, 2008a). There are several factors which may enhance the EVA process such as atherosclerosis via intima–media thickening, smoking through increased production of the reactive oxidative species (ROS), hypertension via activation of the renin–angiotensin system and decreased cell proliferation (Bots and Grobbee, 2002; Hansson, 2005; Nilsson, 2008b).

Recently, there is a growing interest in the potential role of a metabolomics approach to determine molecular mechanisms underlying cardiovascular phenotypes including arterial stiffness. Recent studies have highlighted an association between arterial stiffness and fatty acid, lipid, steroid, carbohydrate, and amino acid metabolism (Kim et al., 2013; Menni et al., 2015; Zagura et al., 2015). In this study we identified 4 down-regulated metabolites associated with EVA syndrome: LPC (18:2), LPC (16:0), LPC (18:1), and LPC (18:0).

Circulating levels of LPC are determined by the combination of LPC production, clearance, and degradation. It is speculated that lower levels of LPC may be reflective of increased catabolism and/or clearance from circulation by acyltransferases and phospholipases, mainly phospholipase A2 (PLA2) catalyzing hydrolysis of phosphatidylcholine (Masuda et al., 2015). Conversely, lower levels of LPC in circulation may result from decreased enzymatic activity of lecithin-cholesterol acyltransferase (LCAT) (Matsumoto et al., 2007; Barber et al., 2012; Law et al., 2019). LCAT is a lipoprotein-associated enzyme which plays a key role in the esterification of free cholesterol and the maturation of high density-lipoprotein (HDL) particles. LCAT is also reported to be involved in the intravascular stage of reverse cholesterol transport which is an anti- atherogenic process by which cholesterol is transported to the liver and excreted (Rousset et al., 2009; Law et al., 2019). It has been demonstrated previously that LACT deficiency, also caused by pathogenic alterations in the LCAT gene, may be associated with increased risk of atherosclerosis and coronary artery disease (Duivenvoorden et al., 2011; Meikle et al., 2011).

LPC constitutes a major plasma lipid, which has been underlined as an important cell signaling molecule (Schmitz and Ruebsaamen, 2010). This metabolite is involved in the transport of components of glycerophospholipid between tissues, and acts as a ligand for specific G protein-coupled receptors (Aiyar et al., 2007). LPC constitutes a major phospholipid component of oxidized low-density lipoproteins (Ox-LDL) (Aiyar et al., 2007; Schmitz and Ruebsaamen, 2010). LPCs are widely recognized for their role in cell proliferation and migration, inflammation and oxidative stress (Colles and Chisolm, 2000). This molecule plays a crucial role in the atherogenic processes in the arterial wall and smooth muscle cells, increasing cellular permeability, apoptosis, inhibition of endothelial relaxation and cell proliferation as well as migration (Ceylan et al., 2004; Kougias et al., 2006). The association between a higher local coronary production of LPC and endothelial dysfunction has been also reported (Chai et al., 1996). Additionally, increased plasma levels of a few LPCs have been shown to be linked with age-related changes that are specific for arterial stiffness (Kim et al., 2013).

Generally, higher LPCs are usually shown to be associated with atherosclerosis and cardiovascular phenotypes (Schmitz and Ruebsaamen, 2010; Stegemann et al., 2011; Kim et al., 2013); however in our study we observed that EVA syndrome in the settings of hypertension was characterized by lower levels of LPCs. The finding may be counterintuitive at first, yet in line with other studies showing that specific LPC species may be negatively associated with cardiovascular risk (Meikle et al., 2011; Fernandez et al., 2013; Lee et al., 2013; Ganna et al., 2014; Stegemann et al., 2014). LPC is considered to be a crucial regulator of oxidative stress in the aging aorta (Lavi et al., 2007). The results of the recent study suggest that LPC may lead to the enhancement of oxidative stress in the rat aorta during aging via the production of reactive species and activation of the 5-lipoxygenase pathway (Zou et al., 2009). Additionally, previous metabolomics and transcriptomics-based experiment revealed that LPCs (16:0), (18:0), and (18:1) were significantly elevated in the aortas of apolipoprotein E knockout mice during early atherosclerosis (Schmitz and Ruebsaamen, 2010). However, other recent report showed that some serum LPCs, such as (16:0) and (18:0), were inversely related to cfPWV, heart rate, asymmetric dimethylarginine (ADMA) and ADMA/arginine in patients with symptomatic atherosclerosis as compared to the controls (Paapstel et al., 2018). Additional research and clinical studies are required to reconcile these conflicting results presented in the literature. Although we provide here few preliminary hypothesis linking lower levels of LPCs with EVA, it is impossible to determine whether LPCs are playing a causal role in promoting EVA or are consequence of compensatory mechanisms.

There are a few limitations to this study. First, the reproducibility of 4 selected metabolites was found to be within a relatively wide range (39–65%) which is likely a consequence of a relatively small sample size and variability present in the data. Second, confidence intervals of odds ratios, describing the risk of EVA, are also fairly wide. Thus, the results should be interpreted with caution and a larger sample size is needed to confirm our findings. Further investigation of enzyme activity (especially PLA2 or lecithin cholesterol acyltransferase) would help to assess whether the observed changes in LPCs levels are a consequence or cause of EVA syndrome.

To our knowledge, this is a first study applying LC-MS based untargeted metabolomics to evaluate metabolic signature of EVA in the settings of hypertension. We demonstrate that hypertensive individuals were characterized by the profile of 4 down-regulated LPCs and were at significantly increased risk of EVA. Our finding warrants further analysis to evaluate clinical utility and validity of these metabolites as early predictors of EVA syndrome in hypertensive patients.

## Data Availability Statement

The datasets generated for this study can be found in the MetaboLights # MTBLS1359.

## Ethics Statement

The studies involving human participants were reviewed and approved by Independent Committee of Bioethical Research at the Medical University of Gdansk. The patients/participants provided their written informed consent to participate in this study.

## Author Contributions

KP, RW, OM, RK, KN, and MM contributed conception and design of the study. AS, MC, MH, KP, and KN performed clinical classification and characteristics of the study population. RW, MK, JR-G, and EB performed plasma analytical measurements and raw data processing. ED-W performed the statistical analysis. KP, RW, and ED-W wrote the first draft of the manuscript. All authors contributed to manuscript revision, read and approved the submitted version.

### Conflict of Interest

The authors declare that the research was conducted in the absence of any commercial or financial relationships that could be construed as a potential conflict of interest.
