# Supplementary material for: Metabolomic Signature of Early Vascular Aging (EVA) in Hypertension
Source: Front Mol Biosci. 2020 Feb 7;7:12. doi: 10.3389/fmolb.2020.00012 (PMC7019377; doi:10.3389/fmolb.2020.00012)
Supplement: Supplementary file 1 [file Data_Sheet_1.PDF]

Supplementary Material

1 Supplementary Tables

Table S1 Characteristics according to cluster 1 and cluster 2 (Model 1 with 4 clusters generated).

	**Cluster 1**<br>**n = 107**	**Cluster 2**<br>**n = 38**	***p*-value**
Age (years)	39.3 ± 13.2	54.1 ± 11.1	< .001
BMI (kg/m^2)	28.6 ± 4.4	28.9 ± 4.1	.717
Male sex *n (%)*	89 (83%)	21 (55%)	< .001
Active smoking *n (%)*	16 (15%)	9 (24%)	.242
cfPWV (m/s)	10.0 ± 2.4	11.1 ± 1.9	.017
Office SBP (mm Hg)	127.6 ± 12.5	136.4 ± 17.7	.001
Office DBP (mm Hg)	71.2 ± 9.6	76.9 ± 11.2	.005
ACE-1/ARB *n (%)*	80 (75%)	38 (100%)	< .001
CCB *n (%)*	46 (43%)	27 (71%)	.003
Beta blockers *n (%)*	48 (45%)	29 (76%)	<.001
Diuretics *n (%)*	39 (36%)	36 (95%)	< .001
Hypolipidemic *n (%)*	62 (58%)	32 (84%)	.002
ASA *n (%)*	16 (15%)	16 (42%)	.001
CVD *n (%)*	6 (6%)	3 (8%)	.624
DM *n (%)*	10 (9%)	13 (34%)	< .001

P-values calculated by t-test or chi-square test. Data presented as mean ± standard deviation or number (and %) for each group. **BMI** – body mass index, **cfPWV** – carotid-femoral pulse wave velocity, **office SBP/DBP** – systolic/diastolic blood pressure measured in a supine position prior to the arterial stiffness examination; **ACE-1/ARB** – angiotensin-converting-enzyme inhibitor/angiotensin II receptor blockers, **CCB** – calcium channel blocker, **diuretics** – thiazides and/or aldosterone antagonists, **hypolipidemic** treatment – statins and/or fibrates, **ASA** – acetyl salicylic acid treatment, **DM** – diabetes mellitus type 2, **CVD** – cardiovascular disease defined as coronary heart disease and/or cerebrovascular disease.

Supplementary Figures

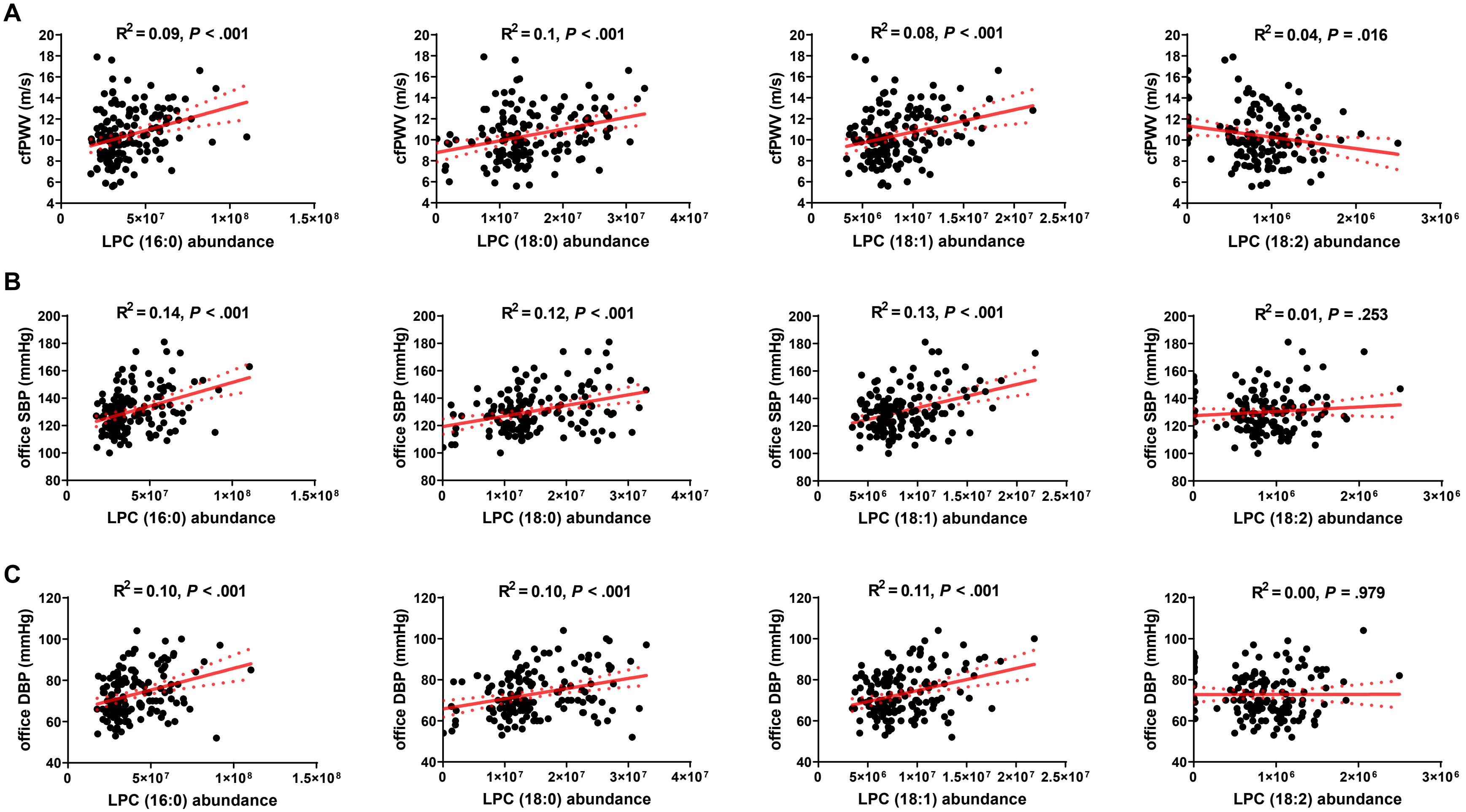

Supplementary Figure 1 Correlations between LPC metabolites and carotid-femoral pulse wave velocity (cfPWV) (**A**), office systolic blood pressure (SBP) (**B**) and office diastolic blood pressure (**C**) in the entire study population.

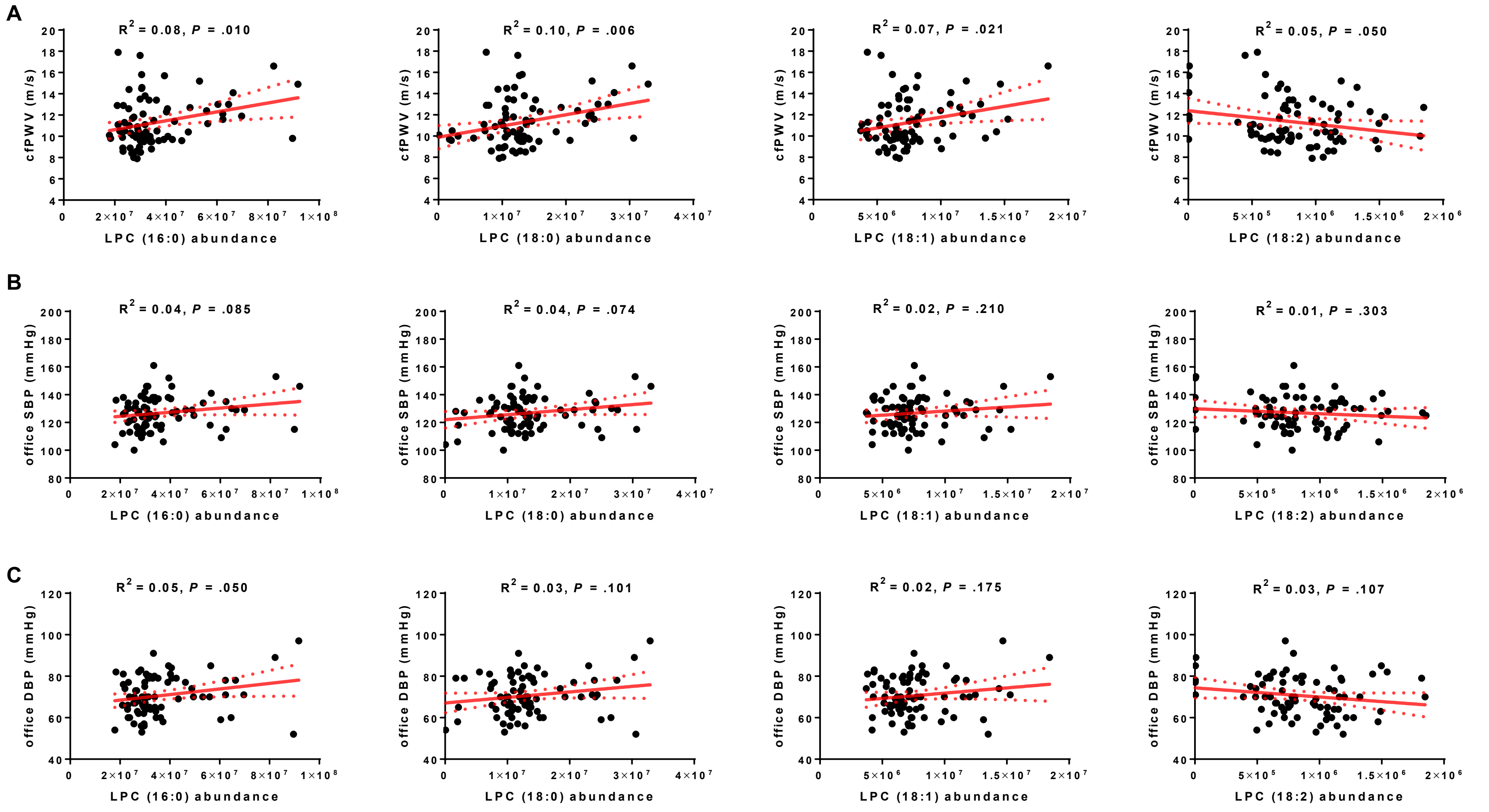

Supplementary Figure 2 Correlations between LPCs and carotid-femoral pulse wave velocity (cfPWV) (**A**), office systolic blood pressure (SBP) (**B**) and office diastolic blood pressure (**C**) in the EVA group.
