# Supplementary material for: Metabolomic Signature of Early Vascular Aging (EVA) in Hypertension
Source: Front Mol Biosci. 2020 Feb 7;7:12. doi: 10.3389/fmolb.2020.00012 (PMC7019377; doi:10.3389/fmolb.2020.00012)
Supplement: Supplementary file 2 [file Data_Sheet_2.PDF]

Neutral mass	RT	Name	Formula	MSI level	Kegg	HMDB	LipidMaps	Metlin	InChIKey	SMILES
**90,0317**	0,7388246	Lactic acid	C3H6O3	3	C01432	HMDB0144295		116	JVTAAEKCZFNVCJ-UHFFFAOYSA-N	CC(O)C(O)=O
**99,0686**	1,4187802	Piperidinone/Hydroxymethylbutanenitrile/Methylpyrrolidinone/Methylpyrrolidone	C5H9NO	3		HMDB0011749		62467	XUWHAWMETYGRKB-UHFFFAOYSA-N	O=C1CCCCN1
**103,0999**	0,6845519	Aminomethylbutanol Valinol	C5H13NO	3				44703	NWYYWIJOWOLJNR-YFKPBYRVSA-N	CC(C)[C@H](CO)N
**103,0999**	7,3616896	Valinol	C5H13NO	3					NWYYWIJOWOLJNR-RXMQYKEDSA-N	CC(C)[C@@H](CO)N
**103,0999**	7,9646416	Aminomethylbutanol/Valinol	C5H13NO	3				44703	NWYYWIJOWOLJNR-YFKPBYRVSA-N	CC(C)[C@H](CO)N
**103,0999**	8,578954	Valinol	C5H13NO	3					NWYYWIJOWOLJNR-RXMQYKEDSA-N	CC(C)[C@@H](CO)N
**106,0417**	1,2566688	Benzaldehyde	C7H6O	3	C00193	HMDB0006115		58358	HUMNYLRZRPPJDN-UHFFFAOYSA-N	O=CC1=CC=CC=C1
**106,0422**	6,5703073	Benzaldehyde	C7H6O	3	C00193	HMDB0006115		58358	HUMNYLRZRPPJDN-UHFFFAOYSA-N	O=CC1=CC=CC=C1
**106,0638**	5,9066973	Trihydroxybutane/Diethylene glycol	C4H10O3	3		HMDB0061944			GTTSNKDQDACYLV-UHFFFAOYSA-N	CCCC(O)(O)O
**113,0585**	0,7145658	Creatinine	C4H7N3O	3	C00791	HMDB0000562		8	DDRJAANPRJIHGJ-UHFFFAOYSA-N	CN1CC(=O)NC1=N
**113,0839**	3,0229337	DMPO	C6H11NO	3				64638		
**117,0791**	0,73305136	Valine/Aminomethylbutyric acid/aminopentanoic acid/Norvaline/Amyl Nitrite/Urea	C5H11NO2	3	C06417				KZSNJWFQEVHDMF-SCSAIBSYSA-N	CC(C)[C@@H](N)C(=O)O
**129,0425**	0,7946795	Pyrrolinehydroxycarboxylic Acid/Acryloylglycine	C5H7NO3	3	C04281	HMDB0062585			WFOFKRKDDKGRIK-DMTCNVIQSA-N	[H][C@@]1(O)C[C@]([H])(N=C1)C(O)=O
**129,0583**	1,979581	Methylene-indolenine	C9H7N	3		HMDB0011664		62407	BCNUXXXHEIUHJB-UHFFFAOYSA-N	C=C1C=NC2=CC=CC=C12
**129,0789**	0,63152486	Acetylaminobutanal/Pipecolic acid/piperidine carboxylic acid/homoproline	C6H11NO2	3	C05936	HMDB0004226		58230	DDSLGZOYEPKPSJ-UHFFFAOYSA-N	CC(=O)NCCCC=O
**129,1514**	5,4255986	Octylamine/Monoctylamine	C8H19N	3	C01740			65641	IOQPZZOEVPZRBK-UHFFFAOYSA-N	CCCCCCCCN
**131,0947**	0,99943894	Diethylglycine/amino-hexanoic acid/Norleucine/Aminocaproic acid/Methylvaline/Leucine/Aminomethylpentanoic acid	C6H13NO2	3	C16647			71280	SGXDXUYKISDCAZ-UHFFFAOYSA-N	CCN(CC)CC(=O)O

**134,0732**	6,5130134	Isochavicol/ t-Anol/Propenylphenol/Hydroxypropenylbenzene/Phenylacetone/Methylacetophenone/Indanol	C9H10O	3	C20464				UMFCIIBZHQXRCJ-NSCUHMNNSA-N	C/C=C/c1ccc(O)cc1
**135,0681**	0,8531394	Phenylacetaldehyde oxime/Phenylacetaldoxime/Phenacylamine/Phenylacetamide	C8H9NO	3	C16075			63615	CXISHLWVCSLKOJ-CLFYSBASSA-N	O/N=C\Cc1ccccc1
**140,0451**	0,6096473	Trifluoromethyl-bismethyl ketone	C5H7F3O	3		HMDB0061928			NLCCAFQWEKMQLM-UHFFFAOYSA-N	CC(C)C(=O)C(F)(F)F
**145,0527**	1,9850607	Indolecarboxaldehyde	C9H7NO	3	C08493	HMDB0029737		67018	OLNJUISKUQQNIM-UHFFFAOYSA-N	O=CC1=CNC2=C1C=CC=C2
**146,1054**	0,6173267	Lysine/Diaminohexanoic acid/diaminohexanoate/Methylornithine	C6H14N2O2	3	C00047	HMDB0000182		25	KDXKERNSBIXSRK-YFKPBYRVSA-N	NCCCC[C@H](N)C(O)=O
**147,0529**	0,743834	lactoyl-Glycine/Glutamic acid/Glutamate/Aminopentanedioic acid/Threomethylaspartate/Methylaspartate/Oxohydroxyaminovalerate/(Carboxymethyl)alanine; Strombine	C5H9NO4	3		HMDB0062183			CLNVOWMDSFOIPV-VKHMYHEASA-N	C[C@H](O)C(=O)NCC(O)=O
**148,0517**	1,2564695	hydroxyphenylpropenal/phenyloxiranecarbaldehyde	C9H8O2	3		HMDB0135274			DCHPWNOJPJRSEA-DUXPYHPUSA-N	[H]\C(C=O)=C(\[H])C1=CC(O)=CC=C1
**155,0351**	0,6876102	Aminopropanol phosphate/Aminopropanyl phosphate/Methylethanolaminium phosphate	C3H10NO4P	3	C04122			63967	YBOLZUJJGUZUDC-GSVOUGTGSA-N	C[C@H](CN)OP(=O)(O)O
**157,0891**	5,3710995	Isoquinoline methanamine	C11H11N	3				45036		
**159,0532**	0,78297263	Methyleneglutamate	C6H9NO4	3	C00651			63273	RCCMXKJGURLWPB-BYPYZUCNSA-N	C=C(C[C@H](N)C(=O)O)C(=O)O
**159,1249**	0,7290204	Aminooctanoic acid	C8H17NO2	3		HMDB0000991	LMFA01100056	74884	AKVBCGQVQXPRLD-UHFFFAOYSA-N	CCCCCCC(N)C(O)=O
**161,1054**	0,7090846	carnitine	C7H15NO3	3	C00318	HMDB0000062		52	PHIQHXFUZVPYII-ZCFIWIBFSA-N	C[N+](C)(C)C[C@H](O)CC([O-])=O
**162,1046**	6,565699	methylphenylbutanone/Tetrahydromethylnaphthalenone/undecadiynal	C11H14O	3		HMDB0133676			YEHRTTZJTORGJL-UHFFFAOYSA-N	CC(CC1=CC=CC=C1)C(C)=O
**165,0786**	1,2572348	Benzyl glycinate/Norsalsolinol/Phenylalanine	C9H11NO2	3		HMDB0059934			JXYACYYPACQCDM-UHFFFAOYSA-N	NCC(=O)OCC1=CC=CC=C1
**166,0631**	7,3584156	Phenyllactic acid	C9H10O3	3	C05607	HMDB0000748		3291	VOXXWSYKYCBWHO-MRVPVSSYSA-N	O[C@H](CC1=CC=CC=C1)C(O)=O

**169,0893**	7,367191	5-Aminoacenaphthene/5-Acenaphthenamine/4-Aminobiphenyl	C12H11N	3	C19319			73029	JEUAWMJVEYFVNJ-UHFFFAOYSA-N	Nc1ccc2c3c(cccc13)CC2
**170,0948**	5,76132	Dendryphiellic acid B/Ipomeadiol	C9H14O3	3			LMFA01020399		KFHQGKGXRZPBOB-YFIBRCQFSA-N	C[C@@H](CCO)/C=C/C=C/C(O)=O
**170,1416**	6,0328884	Acrylamidopropyl trimethylammonium	C9H18N2O	3				34500		
**172,1466**	6,834683	Isocapric acid	C10H20O2	3			LMFA01020247	73686	OAOABCKPVCUNKO-UHFFFAOYSA-N	CC(C)CCCCCCC(O)=O
**174,1118**	0,72224355	Arginine	C6H14N4O2	3	C00792	HMDB0003416		6924	ODKSFYDXXFIFQN-SCSAIBSYSA-N	N[C@H](CCCNC(N)=N)C(O)=O
**176,0476**	6,897944	Epoxy-decenediynoic acid	C10H8O3	3			LMFA01070027		UBMRQHZPEXIXEJ-ALCCZGGFSA-N	CC1OC1C#CC#C/C=CC(O)=O
**180,0787**	6,319259	(methoxyphenyl)propanoic acid/hydroxyphenylbutanoic acid	C10H12O3	3		HMDB0131174			FIUFLISGGHNPSM-UHFFFAOYSA-N	COC1=CC=C(CCC(O)=O)C=C1
**181,0737**	0,794167	Tyrosine; Hydroxyphenylalanine/Threo Phenylserine/Trihydroxytetrahydroisoquinoline	C9H11NO3	3	C00082	HMDB0000158		34	OUYCCCASQSFEME-QMMMGPOBSA-N	N[C@@H](CC1=CC=C(O)C=C1)C(O)=O
**187,0633**	1,9841279	Indoleacrylic acid/indolacrylateIndolylpropenoate	C11H9NO2	3		HMDB0000734		5702	SXOUIMVOMIGLHO-AATRIKPKSA-N	OC(=O)\C=C\C1=CC2=C(N1)C=CC=C2
**188,0797**	0,8245183	Glycyl-Hydroxyproline/Acetylglutamine	C7H12N2O4	3		HMDB0011173		62021	ZJQXGJBINIMBOY-UHFFFAOYSA-N	NCC(=O)N1CC(O)CC1C(O)=O
**190,1361**	7,187217	Megastigmatrienone/dehydro-beta-Ionone/10-fluoro-capric acid	C13H18O	3		HMDB0059906			YKVWPZJHENXDAJ-VOTSOKGWSA-N	[H]\C(C=C)=C(\[H])C1C(C)=CC(=O)CC1(C)C
**192,0275**	0,7592171	didehydrogluconic acid/Isocitric acid; Hydroxypropanetricarboxylate; erythro-Isocitric acid/Diketogulonate/diketogulonic acid/Citric acid /Glucarolactone/Dehydrodeoxyglucarate; Dihydroxyoxohexanedioate/Galactarolactone/Dehydrodeoxyglucarate/Carboxymethyloxysuccinate	C6H8O7	3	C02780		LMFA01050471	65828	RXMWXENJQAINCC-DMTCNVIQSA-N	OC(=O)C(=O)[C@@H](O)[C@H](O)C(=O)CO
**194,0951**	7,3584867	Hydroxyphenyl-valeric acid	C11H14O3	3		HMDB0041666		96026	CMLIEOOXQFWANJ-UHFFFAOYSA-N	OC(=O)CCCCC1=CC=CC(O)=C1
**202,172**	7,0167837	Vetivenene/Curcumene/Aro	C15H22	3		HMDB0059			QSUQBXKPPUWLTH	[H][C@@]1(C)CC=CC2=CCC(C[C@@]12C)=C(C)C

		madendradiene/Calamenene				857			-DOMZBBRYSA-N	
**203,1158**	0,74607474	Acetylcarnitine/Lactoyl-Leucine	C9H17NO4	3		HMDB0000201	LMFA07070050	956	RDHQFKQIGNGIED-MRVPVSSYSA-N	CC(=O)O[C@@H](CC([O-])=O)C[N+](C)(C)C
**204,0789**	8,416487	Levulinic Acid, 3-Benzylidenyl-/3-Butylidene-7-hydroxyphthalide	C12H12O3	3				44382		
**204,0895**	1,9823583	D-Tryptophan	C11H12N2O2	3	C00525	HMDB0013609		65364	QIVBCDIJIAJPQS-SECBINFHSA-N	N[C@H](CC1=CNC2=C1C=CC=C2)C(O)=O
**204,0899**	1,9823583	Hydroxymethylantipyrine/Tryptophan	C11H12N2O2	3		HMDB0013840		85279	JBJKAGOWIZGVTR-UHFFFAOYSA-N	CN1N(C(=O)C=C1CO)C1=CC=CC=C1
**208,0848**	1,2005575	Kynurenine/Anthraniloylalanine/Formylhydroxykynurenamine	C10H12N2O3	3	C00328	HMDB0000684			YGPSJZOEDVAXAB-QMMMGPOBSA-N	N[C@@H](CC(=O)C1=CC=CC=C1N)C(O)=O
**208,1104**	6,7832627	oxo-dodecatrienoic acid/(tert-Butyl-phenoxy)-acetic acid/Cyclohexene-acrylic acid-trimethyl-oxo/(tert-Butyl-phenoxy)-acetic acid/Coronafacic acid	C12H16O3	3			LMFA01060095	74733	ZRIOISYGDYRQIK-JDNZBGOLSA-N	OC(=O)CCC/C=C/C/C=C/C=C\C=O
**213,2454**	6,48777	Tetradecylamine	C14H31N	3				3313		
**217,1314**	0,8019142	Propionylcarnitine	C10H19NO4	3	C03017	HMDB0000824	LMFA07070105	36668	UFAHZIUFPNSHSL-UHFFFAOYSA-N	CCC(=O)OC(CC([O-])=O)C[N+](C)(C)C
**218,0197**	0,68900734	dihydroxyfurochromenone	C11H6O5	3		HMDB0135848			FQNXUBCEYKWIGF-UHFFFAOYSA-N	OC1=CC2=C(O1)C=CC1=C2OC(=O)C=C1O
**218,167**	6,0282784	Octyl phenyl ketone/benzyloctanal/(phenylmethylidene)octanol	C15H22O	3		HMDB0094674			PFUPABFCHVRLLY-UHFFFAOYSA-N	CCCCCCCC(=O)C1=CC=CC=C1
**218,1672**	6,244601	N-Octyl phenyl ketone/2-benzyloctanal/(phenylmethylidene)octanol	C15H22O	3		HMDB0094674			PFUPABFCHVRLLY-UHFFFAOYSA-N	CCCCCCCC(=O)C1=CC=CC=C1
**221,178**	5,8445644	Tapentadol	C14H23NO	3				96332		
**222,0567**	6,6430306	Triethyl-trithiane/(Methylthio)propyl malate	C9H18S3	3		HMDB0040262		94791	PHQSYHLEPPMJSU-UHFFFAOYSA-N	CCC1SC(CC)SC(CC)S1
**224,1888**	6,694408	Anaferine/Anapheline/Dicyclohexylurea	C13H24N2O	3	C06183			64435	JFMCQBGTUJUOAB-RYUDHWBXSA-N	O=C(C[C@@H]1CCCCN1)C[C@@H]1CCCCN1
**225,9452**		Dichlorooxohexenedioate	C6H4Cl2O5	3	C12835	HMDB0060363	LMFA01090152	69492	PLPVRWUZGSFJJB-OWOJBTEDSA-N	OC(=O)/C(/Cl)=CC(=O)C(Cl)C(O)=O
**228,1475**	0,76670134	Leucylproline/Isoleucylproline	C11H20N2O3	3		HMDB0011175			VTJUNIYRYIAIHF-UHFFFAOYSA-N	CC(C)CC(N)C(=O)N1CCCC1C(O)=O
**228,2088**	9,566135	Myristic acid	C14H28O2	3	C06424	HMDB0000806	LMFA01010014	196	TUNFSRHWOTWDNC-UHFFFAOYSA-N	CCCCCCCCCCCCCC(O)=O
**229,1686**	5,679458	Decanoylglycine	C12H23NO	3		HMDB0013			WRRYZYASRAUROW	CCCCCCCCCC(=O)NCC(O)=O

			3			267			-UHFFFAOYSA-N	
**229,2405**	6,25078	Xestoaminol C	C14H31NO	3			LMSP01080033	53933	WMUMHAZHWIUBPN-UONOGXRCSA-N	C[C@@H](N)[C@@H](O)CCCCCCCCCCC
**234,0853**	0,8481127	Ethylenediaminetriacetic acid/Ethylenediaminetriacetate ED3A/Aspartyl-Threonine/Glutamylserine/Aminocarboxyethylglutamate	C8H14N2O6	3	C21396				OUDSFQBUEBFSPS-UHFFFAOYSA-N	O=C(O)CNCCN(CC(=O)O)CC(=O)O
**234,1621**	7,9165196	Valerenic acid	C15H22O2	3	C09743				FEBNTWHYQKGEIQ-SUKRRCERSA-N	CC1=C2[C@H](/C=C(\C)C(=O)O)CC[C@@H](C)[C@H]2CC1
**238,0845**	5,700519	Trimethoxycinnamic acid/Trimethoxycinnamate/dimethoxybenzodioxolpropenol/hydroxytrimethoxyphenylpropenal/dimethoxybenzodioxolpropanal/trimethoxyphenylpropenoic acid/Propanoic acid, hydroxy(propenyloxy)phenoxy	C12H14O5	3		HMDB0002511		6705	YTFVRYKNXDADBI-SNAWJCMRSA-N	COC1=CC(\C=C\C(O)=O)=CC(OC)=C1OC
**240,0996**	6,319	trimethoxyphenylpropanoic acid	C12H16O5	3		HMDB0142074			QOPNYPCVRBRZOP-UHFFFAOYSA-N	COC1=C(OC)C(OC)=C(CCC(O)=O)C=C1
**240,1001**	6,319	(hydroxymethyl)methoxymethyldihydrobenzopyrandiol/(hydroxypropenyl)trimethoxyphenol/(trimethoxyphenyl)propanoic acid/methoxydimethyldihydrobenzopyrantriol/hydroxyhydroxymethoxyphenylpentanoic acid/dimethoxybenzodioxolylpropanol/(hydroxytrimethoxyphenyl)propanal	C12H16O5	3		HMDB0125804			WPDUHZUOYGPEOT-UHFFFAOYSA-N	COC1=CC(O)=C2C(O)CC(C)(CO)OC2=C1
**240,1471**	0,76480293	Pirbuterol	C12H20N2O3	3	C07807	HMDB0015407		66744	VQDBNKDJNJQRDG-UHFFFAOYSA-N	CC(C)(C)NCC(O)C1=NC(CO)=C(O)C=C1
**243,1836**	5,770232	N-Undecanoylglycine	C13H25NO3	3		HMDB0013286			HEUQYIQQCNOXOG-UHFFFAOYSA-N	CCCCCCCCCCC(=O)NCC(O)=O
**246,0854**	0,78740627	Aspartylhydroxyproline/Dihydrouridine	C9H14N2O6	3		HMDB0011160		62009	WJDUWENBTQEKDZ-UHFFFAOYSA-N	NC(CC(O)=O)C(=O)N1CC(O)CC1C(O)=O
**246,1215**	1,1686447	Bis(acetamido)trideoxyaltropyranose/Aspartyl-Leucine/Glutamylvaline/Diacetamidotrideoxymannopyran	C10H18N2O5	3	C19972			73461	NRXWTRNYICXMBF-SGZWNVLDSA-N	CC(=O)N[C@H]1[C@H](C)O[C@H](O)[C@H](NC(C)=O)[C@H]1O

		ose;Diacetamidotrideoxymannopyranose								
**248,1008**	0,7615933	Glutamylthreonine	C9H16N2O6	3		HMDB0028829		85743	JSIQVRIXMINMTA-ZDLURKLDSA-N	C[C@@H](O)[C@H](NC(=O)[C@@H](N)CCC(O)=O)C(O)=O
**248,1782**	7,9236126	Hexadecatetraenoic acid	C16H24O2	3				34833		
**248,199**	6,8372703	alpha-Butyl-omega-hydroxypoly(oxyethylene) poly(oxypropylene)	C13H28O4	3		HMDB0032181		88248	PXWCUJRVSZCPHE-UHFFFAOYSA-N	CCCCOCCOCC(C)OCCCOC
**254,2246**	9,833729	palmitoleic acid	C16H30O2	3	C08362	HMDB0003229	LMFA01030056	188	SECPZKHBENQXJG-FPLPWBNLSA-N	CCCCCC/C=C\CCCCCCCC(O)=O
**256,2404**	10,793896	Palmitic acid	C16H32O2	3	C00249	HMDB0000220	LMFA01010001	187	IPCSVZSSVZVIGE-UHFFFAOYSA-N	CCCCCCCCCCCCCCCC(O)=O
**257,103**	0,7567323	Glycerylphosphorylcholine/ Methylcytidine	C8H20NO6P	3		HMDB0000086		370	SUHOQUVVVLNYQR-QMMMGPOBSA-N	C[N+](C)(C)CCOP([O-])(=O)OC[C@@H](O)CO
**259,1786**	5,5685873	Glutamic acid dibutyl ester/Hexanoylcarnitine	C13H25NO4	3				3545		
**260,1376**	2,8106732	Oxypyrithiamine/Hydroxymethylpyrimidylmethyl hydroxyethyl methylpyridinium/Glutamylleucine	C14H18N3O2	3	C21112				PINBHFPAOLBYDE-UHFFFAOYSA-O	Cc1ncc(C[n+]2cccc(CCO)c2C)c(O)n1
**264,1113**	5,1679993	Alpha Phenylacetyl glutamine	C13H16N2O4	3	C04148	HMDB0006344		58397	JFLIEFSWGNOPJJ-JTQLQIEISA-N	NC(=O)CC[C@H](NC(=O)CC1=CC=CC=C1)C(O)=O
**264,1113**	5,1679993	di-Hydroxymelatonin	C13H16N2O4	3		HMDB0061136			UEZDFVJSDBULCI-UHFFFAOYSA-N	COC1=C(O)C2=C(NC=C2CCNC(C)=O)C=C1O
**264,1113**	5,1679993	Acetylformylmethoxykynurenamine	C13H16N2O4	3	C05642	HMDB0004259		44788	JYWNYMJKURVPFH-UHFFFAOYSA-N	COC1=CC(C(=O)CCNC(C)=O)=C(NC=O)C=C1
**266,1652**	7,8064904	Diphenylheptenol/aminobutylhydroxymethoxyphenyl propanimidic acid/diphenylheptanone	C19H22O	3		HMDB0030146		86754	DPRCKWANIKZGTF-QPEQYQDCSA-N	OC(CC\C=C/C1=CC=CC=C1)CCC1=CC=CC=C1
**266,261**	11,317516	octadecenal	C18H34O	3		HMDB0030965	LMFA06000099	75373	ZENZJGDPWWLORF-MDZDMXLPSA-N	CCCCCCCC/C=C/CCCCCCCC=O
**268,1317**	6,78753	carboxy-methyl-pentyl-furanpropanoic acid/DMABA-d6 NHS ester	C14H20O5	3		HMDB0061643			RIJDKRLRDVBUHJ-UHFFFAOYSA-N	[H]OC(=O)C1=C(OC(=C1C([H])([H])[H])C([H])([H])C([H])([H])C([H])([H])C([H])([H])C([H])([H])[H])C([H])([H])C([H])([H])C(=O)O[H]
**268,1661**	7,005603	Prostaglandin lactone-diol/Corey PG-Lactone Diol	C15H24O4	3		HMDB0240213			UMDQPEMZJHAFNL-FNLFQXNBSA-N	[H][C@]12C[C@@H](O)[C@H](\C=C\[C@@H](O)CCCCC)[C@@]1([H])CC(=O)O2
**271,2514**	6,57696	Hexadecanoic acid/aminoAminohexadecanoic acid/C16 Sphingosine	C16H33NO2	3				74869		
**273,267**	6,228155	C16 Sphinganine	C16H35NO2	3	C13915		LMSP01040001	41556	ZKLREJQHRKUJHD-JKSUJKDBSA-N	CCCCCCCCCCCCC[C@@H](O)[C@@H](N)CO

**275,1383**	0,90102667	Glutarylcarnitine	C12H21NO6	3		HMDB0013130			NXJAXUYOQLTISD-SECBINFHSA-N	C[N+](C)(C)C[C@@H](CC([O-])=O)OC(=O)CCCC(O)=O
**278,0866**	6,5708075	(2-Chlorophenyl)diphenylmethane/Triphenylphosphine oxide/11-bromo-dodecanoic acid	C19H15Cl	3				1898		
**278,1521**	8,4037	dimethyloxiranylmethylpentenyllbenzenetriol	C16H22O4	3		HMDB0133086			JYHLZRSNMLULIG-UHFFFAOYSA-N	CC(CCC1OC1(C)C)=CCC1=C(O)C=C(O)C=C1O
**278,2247**	7,7563353	Linolenic acid	C18H30O2	3	C06427	HMDB0001388	LMFA01030152	192	DTOSIQBPPRVQHS-PDBXOOCHSA-N	CC/C=C\C/C=C\C/C=C\CCCCCCCC(O)=O
**278,2247**	9,395291	Linolenic acid	C18H30O2	3	C06427	HMDB0001388	LMFA01030152	192	DTOSIQBPPRVQHS-PDBXOOCHSA-N	CC/C=C\C/C=C\C/C=C\CCCCCCCC(O)=O
**279,0867**	0,6778833	Ser Ala Cys/Gly Thr Cys	C9H17N3O5S	3				15654		
**279,2566**	9,135976	Linoleamide	C18H33NO	3		HMDB0062656	LMFA08010008	43435	SFIHQZFZMWZOJV-HZJYTTRNSA-N	CCCCC/C=C\C/C=C\CCCCCCCC(N)=O
**280,2403**	10,150115	Linoleic acid	C18H32O2	3	C01595	HMDB0000673	LMFA01030120	191	OYHQOLUKZRVURQ-HZJYTTRNSA-N	CCCCC/C=C\C/C=C\CCCCCCCC(O)=O
**281,2722**	10,082904	Oleamide	C18H35NO	3	C19670	HMDB0002117	LMFA08010004	4115	FATBGEAMYMYZAF-KTKRTIGZSA-N	CCCCCCCC/C=C\CCCCCCCC(N)=O
**282,1676**	5,360094	Hexaethylene glycol	C12H26O7	3		HMDB0061822			IIRDTKBZINWQAW-UHFFFAOYSA-N	OCCOCCOCCOCCOCCOCCO
**282,256**	10,967361	vaccenic acid/octadecenoic acid/oleic acid/elaidic acid	C18H34O2	3	C08367	HMDB0003231	LMFA01030077	3407	UWHZIFQPPBDJPM-BQYQJAHWSA-N	CCCCCC/C=C/CCCCCCCCCC(O)=O
**283,2883**	11,3391	Stearamide	C18H37NO	3	C13846	HMDB0034146	LMFA08010003	34494	LYRFLYHAGKPMFH-UHFFFAOYSA-N	CCCCCCCCCCCCCCCCCC(N)=O
**284,2143**	9,365747	Dimethyl-norandrostadienone	C20H28O	3	C14655			70237	SPKAUGWCKWXQFM-QSFXBCCZSA-N	CC1(C)CCC2=C1CC[C@H]1[C@H]2CCC2=CC(=O)CC[C@@]21C
**285,1366**	6,883469	Cherylline	C17H19NO3	3	C12167			69337	VXXVFIKKBBVGIR-HNNXBMFYSA-N	COc1cc2c(cc1O)CN(C)C[C@H]2c1ccc(O)cc1
**285,194**	5,708251	Octenoylcarnitine	C15H27NO4	3		HMDB0013324			YMIVWYONPRZBEJ-LXKVQUBZSA-N	CCCCC\C=C\C(=O)O[C@@H](CCC([O-])=O)[N+](C)(C)C
**287,1957**	5,3906727	Arginyl-Leucine	C12H25N5O3	3		HMDB0028713		85628	WYBVBIHNJWOLCJ-UHFFFAOYSA-N	CC(C)CC(NC(=O)C(N)CCCNC(N)=N)C(O)=O
**287,2095**	5,834523	Octanoylcarnitine	C15H29NO4	3				85173		
**287,2826**	6,359945	C17 Sphinganine	C17H37NO2	3			LMSP01040003	41558	KFQUQCFJDMSIJF-DLBZAZTESA-N	CCCCCCCCCCCCCC[C@@H](O)[C@@H](N)CO
**290,1881**	7,6473913	oxooctadecatetraenoic acid	C18H26O3	3			LMFA02000270	74765	ZYOSGENSARGRME-BEGPLMEHSA-N	CC/C=C/C=C/C=C\C=C/CCCCC(=O)CCC(O)=O
**292,2404**	9,074539	Dihydroxyandrostane	C19H32O2	3			LMST02020093		CBMYJHIOYJEBSB-ULTMTAQRSA-N	C[C@@]12CC[C@@H]3[C@H](CC[C@H]4C[C@@H](O)CC[C@]43C)[C@H]1CC[C@@H]2O

**294,2192**	7,147893	hydroxyoctadecatrienoic acid	C18H30O3	3		HMDB0010203	LMFA02000029	36032	KLLGGGQNRTVBSU-JDTPQGGVSA-N	CC/C=C\CC(O)/C=C/C=C\CCCCCCCC(O)=O
**294,2196**	7,8034964	hydroxy-linolenic acid	C18H30O3	3				45842		
**294,2196**	8,048426	hydroxy-linolenic acid	C18H30O3	3				45842		
**295,0595**	0,6855486	Aminoimidazole ribonucleotide	C8H14N3O7P	3	C03373	HMDB0001235		6097	PDACUKOKVHBVHJ-XVFCMESISA-N	NC1=CN=CN1[C@@H]1O[C@H](COP(O)(O)=O)[C@@H](O)[C@H]1O
**296,2351**	7,7652965	Epoxyoctadecenoic acid/ Hydroxyoctadecadienoic acid	C18H32O3	3	C08368				CCPPLLJZDQAOHD-BEBBCNLGSA-N	CCCCC[C@@H]1O[C@@H]1C/C=C\CCCCCCCC(=O)O
**298,1934**	6,751142	All-TransDidehydro-Retinoic acid/Hydroxy-norpregna-trien-one/Eremolactone/Methoxysecocycloandrostatrienone/Dimethylhexestrol/Norethynodrel/Methoxyhomoestratetraenabeta-ol	C20H26O2	3		HMDB0060092	LMPR01090020	41514	SYESMXTWOAQFET-YCNIQYBTSA-N	CC1C=CCC(C)(C)C=1/C=C/C(/C)=C/C=C/C(/C)=C/C(O)=O
**299,2825**	9,26306	ketosphinganine/Sphingosine	C18H37NO2	3	C02934	HMDB0001480	LMSP01020002	3428	KBUNOSOGGAARKZ-KRWDZBQOSA-N	CCCCCCCCCCCCCCCC(=O)[C@@H](N)CO
**300,2092**	7,411182	Retinoic Acid	C20H28O2	3	C00777	HMDB0001852	LMPR01090019	2277	SHGAZHPCJJPHSC-YCNIQYBTSA-N	CC1CCCC(C)(C)C=1/C=C/C(/C)=C/C=C/C(/C)=C/C(O)=O
**301,2249**	5,893207	dimethylheptanoyl carnitine/Nonanoylcarnitine	C16H31NO4	3		HMDB0006320	LMFA07070029	58391	QBYXBONNCVATNQ-UHFFFAOYSA-N	CC(C)CCCC(C)C(=O)OC(C[N+](C)(C)C)CC([O-])=O
**302,2247**	8,156109	eicosapentaenoic acid	C20H30O2	3			LMFA01030396	74063	IFPCWRDOHFTYMB-BWGKZPQXSA-N	CCCCC/C=C\C/C=C\C/C=C\C/C=C\C/C=C/C(O)=O
**302,2247**	9,263333	eicosapentaenoic acid	C20H30O2	3			LMFA01030396	74063	IFPCWRDOHFTYMB-BWGKZPQXSA-N	CCCCC/C=C\C/C=C\C/C=C\C/C=C\C/C=C/C(O)=O
**302,2248**	8,017053	EPA	C20H30O2	3	C06428	HMDB0001999	LMFA01030759	6423	JAZBEHYOTPTENJ-JLNKQSITSA-N	CC/C=C\C/C=C\C/C=C\C/C=C\C/C=C\CCCC(O)=O
**302,2455**	8,689119	MG(14:0)/3,4-dihydroxy-4-methylhexadecanoic acid	C17H34O4	3		HMDB0011561		62344	DCBSHORRWZKAKO-INIZCTEOSA-N	[H][C@](O)(CO)COC(=O)CCCCCCCCCCCCC
**302,2459**	8,885422	MG(14:0)/3,4-dihydroxy-4-methylhexadecanoic acid	C17H34O4	3		HMDB0011530		62314	TVIMZSOUQXNWHO-UHFFFAOYSA-N	[H]C(CO)(CO)OC(=O)CCCCCCCCCCCCC
**303,205**	5,5893235	hydroxyoctanoyl carnitine	C15H29NO5	3		HMDB0061634			LUBMSOHGUAAXAM-UHFFFAOYSA-N	CCCCCC(O)CC(=O)OC(CC([O-])=O)C[N+](C)(C)C
**303,2927**	6,741168	1-hexadecylpyridinium/Cetylpyridinium	C21H37N	3				34547		
**304,2388**	8,170919	Arachidonic acid	C20H32O2	3	C00219	HMDB0001043	LMFA01030001	193	YZXBAPSDXZZRGB-DOFZRALJSA-N	CCCCC/C=C\C/C=C\C/C=C\C/C=C\CCCC(O)=O
**304,2401**	9,9404125	Arachidonic acid	C20H32O2	3	C00219	HMDB0001043	LMFA01030001	193	YZXBAPSDXZZRGB-DOFZRALJSA-N	CCCCC/C=C\C/C=C\C/C=C\C/C=C\CCCC(O)=O
**308,1973**	9,14654	Trifluorotetradecenyl acetate	C16H27F3O2	3			LMFA07010327	46326	QVAHKGHMXZIUOO-PKNBQFBNSA-N	CC(=O)OCCCCCCCCCC/C=C/CC(F)(F)F

**308,235**	9,36398	methylepoxyoctadecadienoate	C19H32O3	3			LMFA01070012	74809	WJRQEOOGBUSIOJ-OCJIRGAFSA-N	COC(=O)CCCCCCCC1OC1C/C=C/C/C=C/CC
**308,2351**	9,459208	Methylpropylfuranundecanoic acid/methyl epoxyoctadecadienoate/Hexyldimethylfuranheptanoic acid/Dimethylpentylfuranoctanoic acid	C19H32O3	3		HMDB0061646			XZOBJEOEOJXQBF-UHFFFAOYSA-N	[H]C1=C(CCC)OC(CCCCCCCCCCC(O)=O)=C1C
**308,271**	10,554331	Dihomolinoleic acid/eicosadienoic acid/linoleic acid/keteleeronic acid/Dimethyloctadienyl decanoate/Octadecadienyl acetate	C20H36O2	3	C16525	HMDB0005060	LMFA01031043	62964	XSXIVVZCUAHUJO-HZJYTTRNSA-N	CCCCC/C=CC/C=CCCCCCCCCCC(O)=O
**310,1172**	1,2156205	Leu-Ala-OH/Abu-Val-OH/Glutamyltyrosine/Cl-Amidine	C14H18N2O6	3				64986		
**313,2252**	5,9851847	9-Decenoylcarnitine	C17H31NO4	3		HMDB0013205	LMFA07070048		GOOOCIIXFLVRAG-UHFFFAOYSA-N	C[N+](C)(C)CC(CC([O-])=O)OC(=O)CCCCCCCC=C
**315,2411**	6,0916	Decanoylcarnitine	C17H33NO4	3		HMDB0000651	LMFA07070059		LZOSYCMHQXPBFU-UHFFFAOYSA-N	CCCCCCCCCC(=O)OC(CC([O-])=O)C[N+](C)(C)C
**316,2038**	6,6744666	deoxy-PGJ2/Hydroxy-methylandrostenedione	C20H28O3	3	C14717	HMDB0005079	LMFA03010021	36099	VHRUMKCAEVRUBK-GODQJPCRSA-N	CCCCC/C=C/C=C1\[C@@H](C/C=C\CCCC(O)=O)C=CC\1=O
**316,2039**	7,177156	Epoxy-hydroxymethylandrostenone	C20H28O3	3	C15192			70681	BJYNFFFDYZKDTE-NPBKEMFKSA-N	C[C@]1(O)CC[C@H]2[C@@H]3CCC4=CC(=O)CC[C@]4(C)[C@@]34O[C@H]4C[C@@]21C
**316,2402**	9,371267	Pregnenolone	C21H32O2	3		HMDB0000253		24078	ORNBQBCIOKFOEO-STZXPNGSSA-N	[H][C@@]12CC[C@H](C(C)=O)[C@@]1(C)CC[C@@]1([H])C2CC=C2C[C@H](O)CC[C@]12C
**318,2172**	7,8977923	HEPE	C20H30O3	3		HMDB0012611	LMFA03070054		LRWYBGFSVUBWMO-NQZHIPPKSA-N	CC[C@@H](O)/C=C/C=CC/C=C/C/C=CC/C=CCCCC(O)=O
**318,2191**	7,3433642	HEPE/EpETE	C20H30O3	3		HMDB0010209	LMFA03070053	61547	UDXLGBLAJBYLSZ-XBCQTNLFSA-N	CC/C=CC(O)C/C=C/C=CC/C=CC/C=CCCCC(O)=O
**318,2192**	8,442302	15-HEPE/oxoETE	C20H30O3	3		HMDB0010209	LMFA03070053	61547	UDXLGBLAJBYLSZ-XBCQTNLFSA-N	CC/C=CC(O)C/C=C/C=CC/C=CC/C=CCCCC(O)=O
**318,2193**	6,8250985	HEPE/Oxo-ETE	C20H30O3	3		HMDB0012611	LMFA03070054		LRWYBGFSVUBWMO-NQZHIPPKSA-N	CC[C@@H](O)/C=C/C=CC/C=C/C/C=CC/C=CCCCC(O)=O
**318,2196**	7,0328355	5-Oxo-ETE/5S-HEPE	C20H30O3	3	C14732	HMDB0010217	LMFA03060011	3844	MEASLHGILYBXFO-XTDASVJISA-N	CCCCC/C=C\C/C=C\C/C=C\C=C\C(=O)CCCC(O)=O
**320,2351**	8,165472	Epoxyeicosatrienoic acid/HETE	C20H32O3	3		HMDB0004673	LMFA03090011		DXOYQVHGIODESM-ATELOPIESA-N	CCCCC/C=C/CC1OC1C/C=C/C/C=C/CCCC(O)=O
**323,2099**	8,402247	oxo-tetradecenoyl-L-Homoserine lactone	C18H29NO4	3				45495		
**324,209**	7,9583597	Etonogestrel/Docosatetraynoic acid/Fluoro-B-	C22H28O2	3		HMDB0014439		1954	GCKFUYQCUCGESZ-BPIQYHPVSA-N	[H][C@@]12CC[C@@](O)(C#C)[C@@]1(CC)CC(=C)[C@]1([H])[C@@]3([H])CCC(=O)C=C3CC[C@@]21[H

		hydroxyandrosterone								]
**324,2109**	7,7723227	Etonogestrel/Docosatetraynoic acid/Fluoro-B-hydroxyandrosterone	C22H28O2	3		HMDB0014439		1954	GCKFUYQCUCGESZ-BPIQYHPVSA-N	[H][C@@]12CC[C@@](O)(C#C)[C@@]1(CC)CC(=C)[C@]1([H])[C@@]3([H])CCC(=O)C=C3CC[C@@]21[H]
**327,2407**	6,07759	nitrooctadecenoic acid	C18H33NO4	3		HMDB0062737	LMFA01120003	74886	WRADPCFZZWXOTI-BMRADRMJSA-N	CCCCCCCC/C(=C\CCCCCCCC(O)=O)/[N+]([O-])=O
**328,2406**	9,716304	DHA	C22H32O2	3	C06429	HMDB0002183	LMFA01030185	3457	MBMBGCFOFBJSGT-KUBAVDMBSA-N	CC/C=C\C/C=C\C/C=C\C/C=C\C/C=C\C/C=C\CCC(O)=O
**330,2772**	9,9282255	MG(16:0)/Monopalmitin	C19H38O4	3		HMDB0011564	LMGL01010009	3855	QHZLMUACJMDIAE-SFHVURJKSA-N	CCCCCCCCCCCCCCCC(=O)OC[C@@H](O)CO
**330,278**	10,095161	MG(16:0)	C19H38O4	3		HMDB0011564	LMGL01010009	3855	QHZLMUACJMDIAE-SFHVURJKSA-N	CCCCCCCCCCCCCCCC(=O)OC[C@@H](O)CO
**332,1379**	5,876426	bromohexadecenoic acid	C16H29BrO2	3			LMFA01090012	74830	GPTUUMAGTKZYRF-HNQUOIGGSA-N	OC(=O)CCCCCCC/C=C/CCCCCCBr
**332,2328**	9,071385	Dihydroxypregneone/dihydroxyhexanorvitamin D3 / dihydroxyhexanorcholecalciferol	C21H32O3	3		HMDB0033918		89617	OVQSVMZESAVYPN-UHFFFAOYSA-N	CC(O)C1CC(O)C2C3CCC4=CC(=O)CCC4(C)C3CCC12C
**332,2351**	7,655438	Hydroxypregnenolone	C21H32O3	3	C05138			41866	JERGUCIJOXJXHF-BZXJGPHVSA-N	CC(=O)[C@@]1(O)CCC2[C@@H]3CC=C4C[C@@H](O)CC[C@]4(C)[C@H]3CC[C@@]21C
**334,2122**	7,2205687	oxoHETE	C20H30O4	3		HMDB0062423			MLZJFLKEKVDNAZ-DJEFXLHGSA-N	[H]\C(CCCCC)=C(/[H])C[C@]([H])(O)C([H])=C([H])C(\[H])=C(\[H])/C(/[H])=C(\[H])C(=O)CCCC(O)=O
**334,213**	7,0759716	oxo-HETE/HpEPE	C20H30O4	3		HMDB0062423			MLZJFLKEKVDNAZ-DJEFXLHGSA-N	[H]\C(CCCCC)=C(/[H])C[C@]([H])(O)C([H])=C([H])C(\[H])=C(\[H])/C(/[H])=C(\[H])C(=O)CCCC(O)=O
**336,2299**	7,0165515	DiHETE	C20H32O4	3		HMDB0062749			UXGXCGPWGSUMNI-XGASFZMTSA-N	[H]\C(C\C([H])=C(\[H])C([H])=C([H])[C@@]([H])(O)CCCC(O)=O)=C(/[H])C([H])=C([H])[C@]([H])(O)CCCCC
**337,3352**	12,262428	Docosenamide	C22H43NO	3				64926		
**340,2044**	7,141521	Hydroxyoxopregnadienecarboxylic acid, lactone/D1-2-Hydroxymethylethisterone/Canrenone	C22H28O3	3	C15090			70583	XFDFDWJXWMKQGW-WNHSNXHDSA-N	C[C@]12C=CC(=O)C=C1CC[C@@H]1[C@@H]2CC[C@@]2(C)[C@H]1CC[C@@]21CCC(=O)O1
**340,2585**	9,11309	HpEDE	C20H36O4	3			LMFA01040030	35361	KEXNVBSLXJLOPR-XMSPSUPSSA-N	CCCCC[C@@H](/C=C/C=C\CCCCCCCCC(O)=O)OO
**342,2193**	7,575176	Hydroxymethylethisterone/epoxy-DHA	C22H30O3	3		HMDB0060710			JGJFMZZHCOKXHX-RIONBYPISA-N	C[C@]12CC[C@H]3[C@@H](CCC4=CC(=O)[C@H](CO)C[C@]34C)[C@@H]1CCC2(O)C#C
**343,2725**	6,381998	Dodecanoylcarnitine/palmitoyl serine	C19H37NO4	3		HMDB0002250	LMFA07070062	34490	FUJLYHJROOYKRA-QGZVFWFLSA-N	CCCCCCCCCCCC(=O)O[C@@H](CC([O-])=O)C[N+](C)(C)C
**346,1585**	6,110716	Gly Arg Asp	C12H22N6O6	3				16016		
**348,2044**	9,939063	Roxatidine acetate	C19H28N2O4	3		HMDB0015695		85590	SMTZFNFIKUPEJC-UHFFFAOYSA-N	CC(=O)OCC(=O)NCCCOC1=CC=CC(CN2CCCCC2)=C1
**348,2273**	10,154989	Dihydroxypregnanedione	C21H32O4	3	C145			70191	AYEXSTRNLGYBSQ-	CC(=O)[C@@]1(O)CC[C@H]2[C@@H]3CC[C@@H]

		/Hydroxyketopregnanolone			98				YOBHSKELSA-N	4C[C@H](O)CC[C@]4(C)[C@H]3C(=O)C[C@@]21C
**348,2283**	8,156172	Trihydroxypregnenone	C21H32O4	3				84296		
**350,2091**	6,102922	Prostaglandin E3/oxo-leukotriene B4/Oxo-lipoxin A4/oxodihydroxyeicosatetraenoic acid	C20H30O5	3				45612		
**350,2091**	6,309252	Epoxyhydroxyoxoprostadienoate/HEPE/Prostaglandin E3	C20H30O5	3	C04835				YCLHGWBUIYKBPM-NBFHXWIWSA-N	CCCCCC(=O)/C=C/[C@H]1[C@H](O)C[C@@H]2O/C(=C/CCCC(=O)O)C[C@@H]21
**352,2228**	6,224995	hydroperoxy-HETE/hydroxy LTB4/keto-PGF2	C20H32O5	3		HMDB0062798			AZHSPOPOMNJPFD-HRIRBLAHSA-N	[H]\C(C\C([H])=C(\[H])C([H])=C([H])C(CCCC(O)=O)OO)=C(/[H])C([H])=C([H])C(O)CCCCC
**354,2749**	8,647746	MG(18:2)/Linoleoyl Glycerol/PGF2 methyl ether/Ceriporic acid B	C21H38O4	3		HMDB0011568			WECGLUPZRHILCT-GSNKCQISSA-N	[H][C@](O)(CO)COC(=O)CCCCCCC\C=C/C\C=C/CCCCC
**354,2772**	9,474519	MG(18:2)/PGF2 methyl ether/Ceriporic acid B/Linoleoyl Glycerol	C21H38O4	3			LMFA03010073	36144	VWEVSUSNDAHANL-GPPPFWBLSA-N	CCCCC[C@@H](O)/C=C/[C@H]1[C@H](C/C=C\CCCCOC)[C@H](O)C[C@@H]1O
**356,1969**	6,5716314	Estradiol diacetate/Dioxopregnadienoic acid methyl ester/Nitenin	C22H28O4	3				43494		
**356,197**	6,8928103	Estradiol diacetate/Dioxopregnadienoic acid methyl ester	C22H28O4	3				43494		
**356,2335**	9,368629	Arachidonyltrifluoromethane/AACOCF3/Estradiol valerate/14-HDoHE/16-Dehydropregnenolone acetate/ 3beta-Acetyloxy-pregna-5,16-dien-20-one	C21H31F3O	3	C01397			63007	PLWROONZUDKYKG-DOFZRALJSA-N	CCCCC/C=C\C/C=C\C/C=C\C/C=C\CCCC(=O)C(F)(F)F
**356,2717**	7,200472	Tetracosahexaenoic acid/Docosahexaenoic Acid ethyl ester	C24H36O2	3				6430		
**357,2878**	6,4788375	palmitoyl threonine	C20H39NO4	3			LMFA08020107	75489	JOIXCEREMHWULC-MJGOQNOKSA-N	C[C@@H](O)[C@H](NC(=O)CCCCCCCCCCCCCCC)C(O)=O
**358,2123**	7,3337665	Piperoic acid	C22H30O4	3		HMDB0040619		95097	VWHKYMBCXCSQEZ-KKYJLSSQSA-N	CC(C)=CCC\C(C)=C/CC\C(C)=C\CC1=CC(=CC(O)=C1O)C(O)=O
**358,2135**	6,817359	Canrenoate	C22H30O4	3				1469		
**358,3082**	11,087643	MG(0:0/18:0/0:0)	C21H42O4	3		HMDB0011535		62319	YQEMORVAKMFKLG-UHFFFAOYSA-N	[H]C(CO)(CO)OC(=O)CCCCCCCCCCCCCCCCC
**358,3089**	11,316808	MG(0:0/18:0/0:0)	C21H42O4	3		HMDB0011535		62319	YQEMORVAKMFKLG-UHFFFAOYSA-N	[H]C(CO)(CO)OC(=O)CCCCCCCCCCCCCCCCC
**362,2091**	6,028	Hydroxycorticosterone/Trihydroxypregnenedione/cortisol	C21H30O5	3	C15034			70528	WPZNCXVXQMWZQE-FDNLDUMUSA-N	C[C@]12C[C@H](O)[C@H]3[C@@H](CCC4=CC(=O)CC[C@@]43C)[C@@H]1C[C@@H](O)[C@@H]2C(=

		/trihydroxyoxohexanorvitamin D3 / trihydroxyoxohexanorcholecalciferol								O)CO
**364,1289**	8,133217	Dimethoxydimethylphenylbenzodipyranone/Carpachromene dimethyl ether	C22H20O5	3			LMPK12111650	50067	IMHUWBJRQHCKTE-UHFFFAOYSA-N	COC1C(=O)c2c(OC=1c1ccccc1)c1C=CC(C)(C)Oc1cc2OC
**364,2016**	7,820534	(Dimethylpropenyl)(methylbutenyl)xanthyletin/Fluorodihydroxypregnenedione	C24H28O3	3		HMDB0030730		87156	PIOKBHKPGZHPHS-UHFFFAOYSA-N	CC(C)=CCC1=C2OC(=O)C(=CC2=CC2=C1OC(C)(C)C=C2)C(C)(C)C=C
**364,2016**	7,9646416	Fluorodihydroxypregnenedione/Fluorodihydroxyprogesterone/Dimethylpropenyl)(methylbutenyl)xanthyletin	C21H29FO4	3	C15172			70662	AIFBIDNFLLKTLI-RSBWHNKUSA-N	CC(=O)[C@@]1(O)CC[C@H]2[C@@H]3C[C@H](F)C4=CC(=O)CC[C@]4(C)[C@H]3[C@@H](O)C[C@@]21C
**366,2044**	6,08435	ox-LGD2/carboxy-LTB4	C20H30O6	3			LMFA03100037	96929	RAQOIYLDXSAKSS-DPLATEDBSA-N	CCCCC[C@@H](O)/C=C/C1=C(C/C=C\CCCC(O)=O)C(=O)OC1(C)O
**367,2719**	6,359678	Tetradecadiencarnitine	C21H37NO4	3		HMDB0013331			HXOGMKPCIDSSKJ-NKBLVAAJSA-N	CCCCCCCC\C=C\C=C\CC(=O)O[C@H]([N+](C)(C)C)CC([O-])=O
**368,1662**	6,476347	Testosterone sulfate/Dehydroepiandrosterone sulfate/Hydroxyandrostenone sulfate/Epitestosterone sulfate	C19H28O5S	3		HMDB0002833	LMST05020032	3558	WAQBISPOEAOCOG-DYKIIFRCSA-N	C[C@@]12CC[C@@H]3[C@H](CCC4=CC(=O)CC[C@]43C)[C@H]1CC[C@H]2OS(O)(=O)=O
**368,2331**	10,123493	Oxocholatrienoic Acid	C24H32O3	3			LMST04010429	84733	GFECBJJQSLNADT-IHMUCKAYSA-N	C[C@@H](CCC(O)=O)[C@@H]1CC[C@@H]2[C@H]3C=CC4=CC(=O)C=C[C@@]4(C)[C@@H]3CC[C@]21C
**368,3442**	11,223261	Deoxyvitamin D3	C27H44	3			LMST03020618	42544	FXKMDILIEQILCA-VDVRBEGSSA-N	CC(C)CCC[C@@H](C)[C@H]1CC[C@H]2/C(/CCC[C@@]21C)=C/C=C1/CCCCC/1=C
**369,288**	6,5254145	OleoylSerine/Tetradecenoylcarnitine	C21H39NO4	3				45444		
**370,1815**	6,697586	5a-Dihydrotestosterone sulfate/Epiandrosterone sulfate	C19H30O5S	3			LMST05020023	57975	KYVPWJSGFKNNLD-UXMGNZLBSA-N	C[C@@]12CC[C@@H]3[C@H](CC[C@@H]4CC(=O)CC[C@]43C)[C@H]1CCC2OS(O)(=O)=O
**370,2123**	7,401573	phenyl trinordihydro Prostaglandin A2	C23H30O4	3				45409		
**371,3033**	6,7111597	(±)-Myristoylcarnitine/O-tetradecanoylcarnitine/N-stearoyl serine/Tetradecanoylcarnitine	C21H41NO4	3				85175		
**372,192**	6,029889	Methylprednisone/clavulolactone/phenyl-tetranor-PGE2	C22H28O5	3				1236		
**372,2276**	7,563183	Hydroxyprogesterone	C23H32O4	3				1093		

		acetate								
**372,2283**	8,083069	Epoxyoxopregnenyl acetate/Hydroxyprogesterone acetate/AcetoxypregnEnDione/Deoxycorticosterone acetate/Hydroxypregnenedioneacetate/Carboxy-tocotrienol	C23H32O4	3	C14635			70223	YRLVTBXOGJMZNC-QHKUYLHMSA-N	CC(=O)O[C@H]1CC[C@@]2(C)C(=CC[C@H]3[C@@H]4C[C@H]5O[C@@]5(C(C)=O)[C@@]4(C)CC[C@@H]32)C1
**374,2429**	8,717892	Androstenediol diacetate/Diacetoxyandrostene/Calcitroic acid /hydroxytetranorvitamin D3 carboxylic acid	C23H34O4	3	C15423			70892	FKDRTPPOFBKQAT-KVWWBZGASA-N	CC(=O)O[C@H]1CC[C@H]2[C@@H]3CCC4=C[C@H](OC(C)=O)CC[C@]4(C)[C@H]3CC[C@]12C
**375,3113**	10,080096	Adrenoyl-EA	C24H41NO2	3	C13829	HMDB0013626	LMFA08040047	3725	FMVHVRYFQIXOAF-DOFZRALJSA-N	CCCCC/C=C\C/C=C\C/C=C\C/C=C\CCCCCC(=O)NCCO
**378,2748**	10,213872	Norursodeoxycholic acid	C23H38O4	3			LMST04060021		QYYDXDSPYPOWRO-JHMCBHKWSA-N	C[C@@H](CC(O)=O)[C@@H]1CC[C@@H]2[C@@H]3[C@@H](CC[C@]21C)[C@]1(C)CC[C@H](O)C[C@@H]1C[C@H]3O
**379,2486**	6,9456434	Sphingosine phosphate/palmitoyl-phosphoethanolamine/	C18H38NO5P	3	C06124	HMDB0000277	LMSP01050001	3891	DUYSYHSSBDVJSM-KRWOKUGFSA-N	CCCCCCCCCCCCC/C=C/[C@@H](O)[C@@H](N)COP(O)(O)=O
**380,019**	0,68728715	Thioxanthine-monophosphate/Thioxanthylic acid	C10H13N4O8PS	3	C16618	HMDB0060418		71265	WMRIOGFRJLQENF-UUOKFMHZSA-N	O[C@@H]1[C@@H](COP(O)(O)=O)O[C@H]([C@@H]1O)N1C=NC2=C1N=C(O)N=C2S
**380,1966**	6,7421584	PA(14:1)/Fludrocortisone	C17H33O7P	3		HMDB0062311	LMGP10050038	82357	IQSHJASKBUVXAG-OGZRUICASA-N	CCCC/C=C\CCCCCCCC(=O)OC[C@@H](O)COP(O)(O)=O
**381,2643**	7,071735	Sphinganine-phosphate	C18H40NO5P	3	C01120	HMDB0001383	LMSP01050002	3512	YHEDRJPUIRMZMP-ZWKOTPCHSA-N	CCCCCCCCCCCCCCC[C@@H](O)[C@@H](N)COP(O)(O)=O
**382,1088**	0,70761234	Deoxyaklanonic acid	C21H18O7	3	C12420			63798	GOOWFUBMXUJYPR-UHFFFAOYSA-N	CCC(=O)CC(=O)c1c(CC(=O)O)cc2c(c1O)C(=O)c1c(O)cccc1C2
**382,2127**	7,3865623	LPA(14:0)/Dioxocholatrienoic Acid	C17H35O7P	3		HMDB0114765			QWRVNPWTRIUQKD-UHFFFAOYSA-N	CC(C)CCCCCCCCCCC(=O)OCC(O)COP(O)(O)=O
**387,2464**	5,478015	Lys Gln Leu/linoleoyl taurine	C17H33N5O5	3				19534		
**388,1871**	6,0318613	dihydroxyhydroxypropylmethylbutenylmethylbutanoylchromenone/phenoxy tetranor Prostaglandin E2/ethyl(pentanyl)trihydroxy(hydroxymethyl)tetrahydropyranyl)pyrimidinetrione	C22H28O6	3		HMDB0132234			LVYGPLHUPURZDF-UHFFFAOYSA-N	CCC(C)C(=O)C1=C(O)C(CC=C(C)C)=C(O)C2=C1OC(=O)C=C2C(O)CC
**388,2231**	7,2913384	phenyl trinor PGF2a	C23H32O5	3				45710		

**390,2018**	6,110873	phenoxy tetranor PGF2a	C22H30O6	3				45692		
**390,202**	6,110873	trifluoro-LTB4/Leu Arg Cys/phenoxy tetranor PGF2a	C20H29F3O4	3			LMFA03020019	36250	YKIRCSCMMMEEDI-QAASZIRWSA-N	OC(=O)CCC[C@@H](O)/C=C\C=C\C=C\[C@@H](O)C/C=C\CCCCC(F)(F)F
**392,2175**	6,1693935	Phe Ala Arg/dodecatetraenyldioxolanylhydroperoxyheptenoic acid	C18H28N6O4	3				17594		
**394,2146**	10,832013	dimethyloctadientrihydroxyphenylphenylpropanone/Dihydrodexamethasone	C25H30O4	3		HMDB0133093			XZHQFEUSMBHNND-UHFFFAOYSA-N	CC(C)=CCCC(C)=CCC1=C(O)C(C(=O)CCC2=CC=CC=C2)=C(O)C=C1O
**394,2696**	8,254971	Prostaglandin E2 isopropyl ester	C23H38O5	3				44811		
**396,2282**	7,6592965	PA(15:0)/hydroxy-(phosphonooxy)octadecanoic acid/CG 4305/CG 4305	C18H37O7P	3		HMDB0062324	LMGP10050037	82356	RZDCKQARKXMIQI-QGZVFWFLSA-N	CCCCCCCCCCCCCCC(=O)OC[C@@H](O)COP(O)(O)=O
**396,2291**	7,487876	PA(15:0)	C25H32O4	3	C14642			43240	UDKABVSQKJNZBH-DWNQPYOZSA-N	C=C1C[C@H]2[C@@H]3C=C(C)C4=CC(=O)CC[C@]4(C)[C@H]3CC[C@]2(C)[C@@]1(OC(C)=O)C(C)=O
**398,2069**	6,756163	Trioxocholadienoic Acid	C24H30O5	3			LMST04010365	84672	OWMHJRCOYHXTBV-UJRQNWHJSA-N	C[C@@H](CCC(O)=O)[C@@H]1CC[C@@H]2[C@@H]3[C@@H](CC(=O)[C@]21C)[C@]1(C)C=CC(=O)C=C1CC3=O
**398,2081**	6,162063	Beraprost/Taprostene/Trioxocholadienoic Acid	C24H30O5	3				45629		
**398,2127**	6,5254145	Pregnanalone sulfate (Allopregnanolone sulfate)/Trioxocholadienoic Acid	C21H34O5S	3				3560		
**399,335**	7,092093	Palmitoylcarnitine	C23H45NO4	3	C02990	HMDB0000222	LMFA07070004	36667	XOMRRQXKHMYMOC-OAQYLSRUSA-N	CCCCCCCCCCCCCCCC(=O)O[C@@H](CC([O-])=O)C[N+](C)(C)C
**404,2171**	6,5185633	Cortisol acetate/adonitoxigenin/phenoxy tetranor PGF2a methyl ester/phenoxy trinor Prostaglandin F2a	C23H32O6	3	C02821		LMST02030093	41869	ALEXXDVDDISNDU-JZYPGELDSA-N	CC(=O)OCC(=O)[C@@]1(O)CC[C@H]2[C@@H]3CCC4=CC(=O)CC[C@]4(C)[C@H]3[C@@H](O)C[C@]12C
**408,2123**	5,9047856	Tyr Ala Arg/Arg Ser Phe	C18H28N6O5	3				16793		
**414,2044**	7,0528126	Estratrienetriol triacetate/His Met Lys	C24H30O6	3	C15382			70852	LMXGPAGGDXVARU-ULHSUKCYSA-N	CC(=O)Oc1ccc2c(c1)[C@@H](OC(C)=O)C[C@@H]1[C@@H]2CC[C@]2(C)[C@@H](OC(C)=O)CC[C@@H]12
**416,2151**	10,154117	Gln Arg Asn	C15H28N8O6	3				19732		
**420,2465**	10,122856	Arg Phe Val	C20H32N6O4	3				16471		
**421,3184**	11,3297205	linolenyl carnitine	C25H43NO4	3		HMDB0006319		58390	DFVGGGHKDAHYIU-UHMZJXMFSA-N	CC\C=C/C\C=C/C\C=C/CCCCCCCC(=O)O[C@H]([N+](C)(C)C)CCC([O-])=O

**422,2276**	6,194133	Phe Arg Thr	C19H30N6O5	3			15663		
**423,3346**	6,9261303	Hexadecadienylcarnitine/Linoelaidyl carnitine/O-linoleoylcarnitine	C25H45NO4	3	HMDB0006469	LMFA07070009	58418	MJLXQSQYKZWZCB-DQFWFXSYSA-N	CCCCC/C=C\C/C=C\CCCCCCCC(=O)O[C@@H](CC([O-])=O)C[N+](C)(C)C
**425,3503**	7,183352	11Z-Octadecenylcarnitine/Oleoyl carnitine/Elaidic carnitine/Vaccenyl carnitine/	C25H47NO4	3	HMDB0013338			HITOYGLMAFIRNI-JCKUYFFHSA-N	CCCCCC\C=C/CCCCCCCCCC(=O)O[C@H]([N+](C)(C)C)CCC([O-])=O
**426,2952**	9,472426	Leupeptin	C20H38N6O4	3			44689		
**427,3671**	7,5383897	Stearoylcarnitine/Acylcarnitine C18:0	C25H49NO4	3	HMDB0000848	LMFA07070008	46553	FNPHNLNTJNMAEE-HSZRJFAPSA-N	CCCCCCCCCCCCCCCCCC(=O)O[C@@H](CC([O-])=O)C[N+](C)(C)C
**430,3071**	7,6004715	Dioxo-cholestanoic acid	C27H42O4	3			43135		
**430,3767**	10,410296	toxisterol3 B1/ethoxydihydrovitamin D3 / ethoxydihydrocholecalciferol/Dimethylcholestendiol/Hydroxymethylmethylcholestenol/hydroxysitosterol/tocopherol	C29H50O2	3		LMST03020441	42370	XLEDFPGAWKXGKL-HADSMPNGSA-N	CC(C)CCC[C@@H](C)[C@H]1CC[C@H]2/C(/CCC[C@@]21C)=C/C=C1\C[C@@H](O)CC[C@@]\1(C)OCC
**437,2906**	7,856079	PE(16:0)	C21H44NO6P	3	HMDB0011152	LMGP02070001	46719	QYTPGOPLNFESQC-NUTQULCTSA-N	CCCCCCCCCCCCCC/C=C\OC[C@@H](O)COP(O)(=O)OCCN
**440,29**	0,86470366	trifluorohydroxynorvitamin/trifluorohydroxynorcholecalciferol/Suillin/Acetoxygeranylgeranyldihydroxybenzene/Carboxytocotrienol	C26H39F3O2	3		LMST03020024	41965	QZGLVEKNRHZOIB-PXKDVQFDSA-N	C[C@@]12CCC/C(=C\C=C3\C[C@H](O)CCC\3=C)/[C@H]1CC[C@H]2[C@@H](C)CCCC(O)C(F)(F)F
**440,2902**	10,597854	trifluorohydroxynorvitamin D3 / trifluorohydroxynorcholecalciferol/Carboxytocotrienol	C26H39F3O2	3		LMST03020024	41965	QZGLVEKNRHZOIB-PXKDVQFDSA-N	C[C@@]12CCC/C(=C\C=C3\C[C@H](O)CCC\3=C)/[C@H]1CC[C@H]2[C@@H](C)CCCC(O)C(F)(F)F
**440,2903**	10,007158	trifluorohydroxynorvitamin D3 /trifluorohydroxynorcholecalciferol/Carboxy-tocotrienol	C26H39F3O2	3		LMST03020024	41965	QZGLVEKNRHZOIB-PXKDVQFDSA-N	C[C@@]12CCC/C(=C\C=C3\C[C@H](O)CCC\3=C)/[C@H]1CC[C@H]2[C@@H](C)CCCC(O)C(F)(F)F
**440,2906**	10,936895	trifluorohydroxynorvitamin D3 / trifluorohydroxynorcholecalciferol/Carboxytocotrienol	C26H39F3O2	3		LMST03020024	41965	QZGLVEKNRHZOIB-PXKDVQFDSA-N	C[C@@]12CCC/C(=C\C=C3\C[C@H](O)CCC\3=C)/[C@H]1CC[C@H]2[C@@H](C)CCCC(O)C(F)(F)F
**446,1922**	5,7208805	Tyr His Gln/Asn Gln Trp/Estrone glucuronide	C20H26N6O6	3			15736		
**451,2706**	7,1423097	PC(O-	C21H42NO	3		LMGP010201	40007	BDCLOBVQLDSEOA-	CC(=O)O[C@@H](COCCCCCCCCCC=C)COP([O-

		13:1)/PE(16:1)/LysoPE(16:1)/ Terpendole G	7P			46		OAQYLSRUSA-N	])(=O)OCC[N+](C)(C)C
**451,3058**	8,302436	PC(O-14:1)/PC(P-14:0)	C22H46NO 6P	3		LMGP010700 01	40397	AAFONNYQRYRPOU -WTYVIMSDSA-N	CCCCCCCCCCCC/C=C/OC[C@@H](O)COP([O-])(=O)OCC[N+](C)(C)C
**451,3066**	8,163846	PC(O-14:1)/PC(P-14:0)	C22H46NO 6P	3		LMGP010700 01	40397	AAFONNYQRYRPOU -WTYVIMSDSA-N	CCCCCCCCCCCC/C=C/OC[C@@H](O)COP([O-])(=O)OCC[N+](C)(C)C
**453,2854**	7,6167297	LysoPC(13:0)/LysoPE(16:0)	C21H44NO 7P	3			40277		
**453,2861**	7,484446	LPC(13:0)/LysoPE(16:0)	C21H44NO 7P	3			45334		
**454,3262**	10,718492	Norcholestanehexol	C26H46O6	3	HMDB0002 157	LMST040200 29	57911	PYLPANOYZCSFOX-UCIVWCSPSA-N	C[C@@H](CCC(O)C(O)CO)[C@@H]1CC[C@@H]2[C@@H]3[C@@H](C[C@@H](O)[C@]21C)[C@]1(C)CC[C@H](O)C[C@@H]1C[C@@H]3O
**456,2644**	0,6178456	bromopentacosadienoic acid/bromomethyltetracosa dienoic acid	C25H45BrO 2	3		LMFA010901 03	96830	VRACWEMNOVUYQ J-YIRKJRPPSA-N	CCCCCCCCCCCCCCC/C=C\CC/C(/Br)=C\CCCC(O)=O
**465,3217**	8,848652	PC(P-15:0)/PE(P-18:0)/PE(O-18:1)/TetraHCA	C23H48NO 6P	3		LMGP010700 03	40399	CORCYSWIDKFRAW-HIVNOOBXSA-N	CCCCCCCCCCCCC/C=C\OC[C@@H](O)COP([O-])(=O)OCC[N+](C)(C)C
**467,3009**	6,8983583	PC(14:0)/PE(17:0)/N-Arachidonoyl tyrosine	C22H46NO 7P	3	HMDB0010 379	LMGP010500 12	40278	VXUOFDJKYGDUJI-OAQYLSRUSA-N	CCCCCCCCCCCCCC(=O)OC[C@@H](O)COP([O-])(=O)OCC[N+](C)(C)C
**467,3012**	7,0025916	PC(O-12:0/2:0)/PC(0:0/14:0)/PC(14:0/0:0)/PE(17:0/0:0)/Arachidonoyl tyrosine	C22H46NO 7P	3		LMGP010200 09	40038	WNCMKZYTLBIUSK-JOCHJYFZSA-N	CCCCCCCCCCCCOC[C@@H](COP([O-])(=O)OCC[N+](C)(C)C)OC(C)=O
**467,3364**	7,4883213	LPC(15:0)/LPE(18:0)	C23H50NO 6P	3		LMGP010600 09	40383	PPXKEQMZYFQYCJ-HSZRJFAPSA-N	CCCCCCCCCCCCCCCCOC[C@@H](O)COP([O-])(=O)OCC[N+](C)(C)C
**475,2731**	7,031012	PE(18:3)/LysoPE(18:3)	C23H42NO 7P	3	HMDB0011 508	LMGP020500 17	62293	QULMFYLWLOKODU -CSLWLMPESA-N	CCCCC/C=C\C/C=C\C/C=C\CCCCC(=O)OC[C@@H](O)COP(O)(=O)OCCN
**476,0961**	0,996174	trihydroxy-hydroxy-hydroxyphenyl)-methoxy-oxo-chromenyl]oxyoxane-carboxylic acid	C22H20O1 2	3	HMDB0133 833			FZMPWQPHUKBBSS -UHFFFAOYSA-N	COC1=CC2=C(C(OC3OC(C(O)C(O)C3O)C(O)=O)=C1)C(=O)C(O)=C(O2)C1=CC(O)=CC=C1
**477,2857**	7,3418517	LysoPE(18:2)	C23H44NO 7P	3	HMDB0011 477	LMGP020500 41	62265	SVRBKLJIDJHADS-USWSLJGRSA-N	CCCCC/C=CC/C=CCCCCCCCC(=O)O[C@@H](COP(O)(=O)OCCN)CO
**477,286**	7,330837	LysoPE(18:2)	C23H44NO 7P	3	HMDB0011 507	LMGP020500 11	62292	DBHKHNGBVGWQJE -USWSLJGRSA-N	CCCCC/C=C\C/C=C\CCCCCCCC(=O)OC[C@@H](O)COP(O)(=O)OCCN
**477,2863**	7,240903	PE(18:2)/LysoPE(18:2)	C23H44NO 7P	3	HMDB0011 507	LMGP020500 11	62292	DBHKHNGBVGWQJE -USWSLJGRSA-N	CCCCC/C=C\C/C=C\CCCCCCCC(=O)OC[C@@H](O)COP(O)(=O)OCCN
**477,3214**	7,510645	LPE(19:1)/LPC(16:2)	C24H48NO 6P	3		LMGP020700 03	46721	QKXTUVCRMVYDML -IPCSYXIDSA-N	CCCCCC/C=C\CCCCCCCCC/C=C\OC[C@@H](O)COP(O)(=O)OCCN
**477,3216**	7,6578	PC(O-16:2)/PE(P-19:1)/PE(P-19:1)	C24H48NO 6P	3			40371		
**479,3011**	7,814056	LysoPE(18:1)/LysoPC(15:1)	C23H46NO 7P	3	HMDB0011 505	LMGP020500 64	62291	WAYKKNOEMJFLDI-KOIKXXGWSA-N	CCCCCC/C=CCCCCCCCCCC(=O)OC[C@@H](O)COP(O)(=O)OCCN

**479,302**	7,7994123	LysoPE(18:1)/LysoPC(15:1)	C23H46NO7P	3				45335		
**479,3372**	7,885849	PC(P-16:0)/PC(O-16:1)	C24H50NO6P	3		HMDB0010407	LMGP01070006	40402	HTZINLFNXLXRBC-CQLBIITFSA-N	CCCCCCCCCCCCCC/C=C\OC[C@@H](O)COP([O-])(=O)OCC[N+](C)(C)C
**481,3164**	7,6690063	PC(15:0)/LysoPE(18:0)/PE(18:0)	C23H48NO7P	3		HMDB0010381	LMGP01050016	40282	RJZVWDTYEWCUAR-JOCHJYFZSA-N	CCCCCCCCCCCCCCC(=O)OC[C@@H](O)COP([O-])(=O)OCC[N+](C)(C)C
**481,3165**	8,521498	LysoPC(15:0)/LysoPE(18:0)	C23H48NO7P	3		HMDB0010381	LMGP01050016	40282	RJZVWDTYEWCUAR-JOCHJYFZSA-N	CCCCCCCCCCCCCCC(=O)OC[C@@H](O)COP([O-])(=O)OCC[N+](C)(C)C
**481,3167**	7,1401525	PC(15:0)/LysoPE(18:0)/PE(18:0)	C23H48NO7P	3		HMDB0010381	LMGP01050016	40282	RJZVWDTYEWCUAR-JOCHJYFZSA-N	CCCCCCCCCCCCCCC(=O)OC[C@@H](O)COP([O-])(=O)OCC[N+](C)(C)C
**481,3167**	7,302617	PC(15:0)/LysoPE(18:0)	C23H48NO7P	3				40379		
**481,317**	8,492235	LysoPC(15:0)/LysoPE(18:0)	C23H48NO7P	3				40283		
**481,3173**	8,016298	PC(15:0)/LysoPE(18:0)/PE(18:0)	C23H48NO7P	3		HMDB0010381	LMGP01050016	40282	RJZVWDTYEWCUAR-JOCHJYFZSA-N	CCCCCCCCCCCCCCC(=O)OC[C@@H](O)COP([O-])(=O)OCC[N+](C)(C)C
**481,3173**	10,053656	LysoPC(15:0)/LysoPE(18:0)	C23H48NO7P	3	C21484	HMDB0011130	LMGP02050001	40775	BBYWOYAFBUOUFP-JOCHJYFZSA-N	CCCCCCCCCCCCCCCCCC(=O)OC[C@@H](O)COP(O)(=O)OCCN
**481,3531**	7,8826065	PC(O-16:0)/Lyso-PAF C-16/Hexadecyllysoglycerophosphocholine/Hexadecyllysoglycerophosphocholine	C24H52NO6P	3	C13903			40386	VLBPIWYTPAXCFJ-UHFFFAOYSA-N	CCCCCCCCCCCCCCCCOCC(O)COP(=O)([O-])OCC[N+](C)(C)C
**484,1057**	9,395986	Tyr-Phe4Cl-OH	C24H21ClN2O7	3				64977		
**484,2826**	8,861693	PG(16:0)/Biotin-XX hydrazide	C22H45O9P	3			LMGP04050008	46733	BVJSKAUUFXBDOB-LEWJYISDSA-N	CCCCCCCCCCCCCCCC(=O)OC[C@@H](O)COP(O)(=O)OC[C@@H](O)CO
**484,3532**	10,349505	Hydroxyvitamin D3 diacetate/Hydroxyprevitamin D3 diacetate/dihydroxymethoxydimethyltetradehydrohomovitamin D3 / dihydroxymethoxydimethyltetradehydrohomocholecalciferol	C31H48O4	3			LMST03020656	42582	JRQYVHHHASBIEU-NQTFQPGQSA-N	CC(C)CCC[C@@H](C)[C@H]1CC[C@H]2/C(/CCC[C@@]21C)=C/C=C1/C[C@H](C[C@H](OC(C)=O)C/1=C)OC(C)=O
**493,3167**	7,1728024	PC(16:1)/PE(19:1)	C24H48NO7P	3			LMGP01050021	40287	LFUDDCMNKWEORN-BREAQWACSA-N	CCCCCC/C=C/CCCCCCCC(=O)OC[C@@H](O)COP([O-])(=O)OCC[N+](C)(C)C
**493,3531**	8,112179	LysoPC(P-17:0)	C25H52NO6P	3			LMGP01070007	40403	JJALPXFJIIZPRV-HFQDTZRISA-N	CCCCCCCCCCCCCCC/C=C\OC[C@@H](O)COP([O-])(=O)OCC[N+](C)(C)C
**493,3531**	8,321449	PC(P-17:0)/PE(P-20:0)	C25H52NO6P	3			LMGP01070007	40403	JJALPXFJIIZPRV-HFQDTZRISA-N	CCCCCCCCCCCCCCC/C=C\OC[C@@H](O)COP([O-])(=O)OCC[N+](C)(C)C
**493,3542**	10,067764	PC(P-17:0)/PE(P-20:0)	C25H52NO6P	3			LMGP01070007	40403	JJALPXFJIIZPRV-HFQDTZRISA-N	CCCCCCCCCCCCCCC/C=C\OC[C@@H](O)COP([O-])(=O)OCC[N+](C)(C)C
**495,3329**	7,657617	LPC(16:0)	C24H50N	2			LMGP01020	40048	HEALIQQDEGDS	CCCCCCCCCCCCCCCCOC[C@@H](COP([O-

			O7P				019		LS-XMMPIXPASA-N	])(=O)OCC[N+](C)(C)C)OC(C)=O
**499,2715**	7,036173	PE(20:5)/LysoPE(20:5)	C25H42NO7P	3		HMDB0011519	LMGP02050027	62303	KCWGBXXAJHVZNN-KOYQJJOGSA-N	CC/C=C\C/C=C\C/C=C\C/C=C\C/C=C\CCCC(=O)OC[C@@H](O)COP(O)(=O)OCCN
**501,2855**	7,337089	LysoPE(20:4)	C25H44NO7P	3		HMDB0011487	LMGP02050051	62275	YWOCITMXHHTBAW-XSQXPFHXSA-N	CCCCC/C=CC/C=CC/C=CC/C=CCCCC(=O)O[C@@H](COP(O)(=O)OCCN)CO
**501,2857**	7,328754	LysoPE(20:4)	C25H44NO7P	3		HMDB0011517	LMGP02050009	46715	ROPRRXYVXLDXQO-XSQXPFHXSA-N	CCCCC/C=C\C/C=C\C/C=C\C/C=C\CCCC(=O)OC[C@@H](O)COP(O)(=O)OCCN
**501,2866**	7,217821	LysoPE(20:4)	C25H44NO7P	3				45331		
**503,3017**	7,5806885	LysoPE(20:3)	C25H46NO7P	3		HMDB0011515	LMGP02050066	62300	YBDFPZJQBYCWBA-WMTBOZPISA-N	CCCCCCCC/C=CC/C=CC/C=CCCCC(=O)OC[C@@H](O)COP(O)(=O)OCCN
**505,3526**	8,526322	/CerP(d26:1)PC(O-18:2)/C-8 Ceramidephosphate/PC(P-18:1)	C26H52NO6P	3				40378		
**505,3528**	7,841896	PC(O-18:2)/C-8 Ceramide-1-phosphate/PC(P-18:1)/CerP(d26:1)	C26H52NO6P	3				40378		
**507,3321**	7,365507	LysoPC(17:1)/LysoPE(20:1)	C25H50NO7P	3		HMDB0011512	LMGP02050020	62297	JEAGLCKGAKACCE-OPSAWKISSA-N	CCCCCCCC/C=C\CCCCCCCCCC(=O)OC[C@@H](O)COP(O)(=O)OCCN
**507,333**	7,365507	LPC(17:1)/LysoPE(20:1)	C25H50NO7P	3			LMGP01050126	76568	LPMGFNAQZPADDZ-FJIRUFBNSA-N	CCCCCCC/C=C\CCCCCCCC(=O)OC[C@@H](O)COP([O-])(=O)OCC[N+](C)(C)C
**507,3687**	8,063368	PC(O-18:1)	C26H54NO6P	3			LMGP01070008	40404	WBOMIOWRFSPZMC-AZGSXVSQSA-N	CCCCCCCCCCCCCCCC/C=C/OC[C@@H](O)COP([O-])(=O)OCC[N+](C)(C)C
**507,3689**	8,855181	PC(O-18:1)/PC(P-18:0)	C26H54NO6P	3			LMGP01060034	76582	HEEMBWYZIJABCR-ZXAYODBVSA-N	CCCCCC/C=C\CCCCCCCCCCOC[C@@H](O)COP([O-])(=O)OCC[N+](C)(C)C
**509,3476**	8,569328	LysoPC(O-17:0)/LysoPE(20:0)/	C25H52NO7P	3		HMDB0011511	LMGP02050012	62296	HEQMDGOBDIGJOC-XMMPIXPASA-N	CCCCCCCCCCCCCCCCCCC(=O)OC[C@@H](O)COP(O)(=O)OCCN
**509,3481**	7,944164	LysoPC(O-17:0)/LysoPE(20:0)	C25H52NO7P	3				40054		
**509,3486**	7,781943	LysoPC(17:0)/LysoPE(20:0)	C25H52NO7P	3		HMDB0012108	LMGP01050024	40290	SRRQPVVYXBTRQK-XMMPIXPASA-N	CCCCCCCCCCCCCCCCC(=O)OC[C@@H](O)COP([O-])(=O)OCC[N+](C)(C)C
**509,3843**	8,885228	PC(O-18:0)/1-octadecylglycero-3-phosphocholine	C26H56NO6P	3		HMDB0011149	LMGP01060014	40388	XKBJVQHMEXMFDZ-AREMUKBSSA-N	CCCCCCCCCCCCCCCCCCOC[C@@H](O)COP([O-])(=O)OCC[N+](C)(C)C
**510,1008**	0,7714881	dioxo-trihydroxy-methoxyphenylpropyl-hydroxyphenoxy-trihydroxyoxane--carboxylic acid	C22H22O14	3		HMDB0128829			XGXCFQBAQZFWHL-UHFFFAOYSA-N	COC1=C(O)C=C(O)C(C(=O)C(=O)CC2=CC(O)=C(OC3OC(C(O)C(O)C3O)C(O)=O)C=C2)=C1O
**510,1008**		Mannopyranosyl glucopyranosylphosphoglycerate	C15H27O17P	3	C19793			73364	JGKAZLJSKYSZED-MQZSKFSESA-N	O=C(O)[C@@H](COP(=O)(O)O)O[C@H]1O[C@H](CO)[C@@H](O)[C@H](O)[C@H]1O[C@H]1O[C@H](CO)[C@@H](O)[C@H](O)[C@@H]1O
**511,3271**	6,2905025	PS(O-18:0/0:0)/Scyphostatin	C24H50NO	3			LMGP030600	78862	LLZPQHOZAQNDCM	CCCCCCCCCCCCCCCCCCOC[C@@H](O)COP(O)(=O)

		A	8P			02		-PKTZIBPZSA-N	OC[C@H](N)C(O)=O
**511,3273**	7,1057754	PS(O-18:0)/Scyphostatin A	C24H50NO8P	3		LMGP03060002	78862	LLZPQHOZAQNDCM-PKTZIBPZSA-N	CCCCCCCCCCCCCCCCCCOC[C@@H](O)COP(O)(=O)OC[C@H](N)C(O)=O
**517,3164**	6,313918	PC(18:3)	C26H48NO7P	3			40303		
**517,3166**	7,0514975	PC(18:3)	C26H48NO7P	3	HMDB0010387	LMGP01050128	61697	MRTUWVDDQVMUCR-ACHCNROVSA-N	CCCCC/C=C\C/C=C\C/C=C\CCCCC(=O)OC[C@@H](O)COP([O-])(=O)OCC[N+](C)(C)C
**517,3167**	6,4401526	PC(18:3)	C26H48NO7P	3			40303		
**519,3327**	7,1977835	PC(18:2)/linoleoylglycerophosphocholine	C26H50NO7P	2	HMDB0010386	LMGP01050035	40301	SPJFYYJXNPEZDW-FTJOPAKQSA-N	CCCCC/C=C\C/C=C\CCCCCCCC(=O)OC[C@@H](O)COP([O-])(=O)OCC[N+](C)(C)C
**519,3336**	7,362615	LPC(18:2)	C26H50NO7P	3	HMDB0062711			LSUXCWJOIAWGOU-FZVXUVPBSA-N	[H]C(CCCCC)=C([H])C\C([H])=C(\[H])CCCCCCCC(=O)O[C@]([H])(CO)COP([O-])(=O)OCC[N+](C)(C)C
**521,3484**	7,700527	PC(O-18:1)/LysoPC(18:1)/PC(P-18:0)/PC(18:1)	C26H52NO7P	3		LMGP01020147	40008	ZOFBAJKPGIIQFN-RASRKNKNSA-N	CCCC/C=C\CCCCCCCCCCCOC[C@@H](COP([O-])(=O)OCC[N+](C)(C)C)OC(C)=O
**521,3489**	7,8368483	LysoPC(18:1)	C26H52NO7P	2		LMGP01030009	40174	MULVVBYNDZRKPA-XCZWIQLXSA-N	CCCCCCCCCCCCCC/C=C\OC[C@@H](COP([O-])(=O)OCC[N+](C)(C)C)OC(C)=O
**523,3642**	8,344322	PE(21:0)/PC(18:0)/enantio-PAF C-16/PAF C-16	C26H54NO7P	3		LMGP02050026	77692	JPTRRYQAYCFMLQ-RUZDIDTESA-N	CCCCCCCCCCCCCCCCCCCC(=O)OC[C@@H](O)COP(O)(=O)OCCN
**523,3646**	8,5379	LysoPC(18:0)	C26H54NO7P	2		LMGP02050026	77692	JPTRRYQAYCFMLQ-RUZDIDTESA-N	CCCCCCCCCCCCCCCCCCCC(=O)OC[C@@H](O)COP(O)(=O)OCCN
**525,2856**	7,2928596	LysoPE(22:6)	C27H44NO7P	3	HMDB0011526	LMGP02050013	62310	XEVRBOQZSXWGQO-PAUXXPOVSA-N	CC/C=C\C/C=C\C/C=C\C/C=C\C/C=C\C/C=C\CCC(=O)OC[C@@H](O)COP(O)(=O)OCCN
**527,3014**	7,466227	LysoPE(22:5)	C27H46NO7P	3	HMDB0011525	LMGP02050070	62309	OSRWIBSZJTWIKA-SCFYABBUSA-N	CC/C=CC/C=CC/C=CC/C=CC/C=CCCCCCC(=O)OC[C@@H](O)COP(O)(=O)OCCN
**528,2007**	7,541605	Physalin	C28H32O10	3	HMDB0039081		93687	QFAOFAWTSOFSQA-UHFFFAOYSA-N	CC1C(=O)OC2CC1(C)C1C(=O)C3(O)OC11C2(C)OC(=O)C1(O)CCC1C3C(O)C=C2CC=CC(=O)C12C
**535,3272**	6,4479384	PC(18:1)	C26H50NO8P	3		LMGP01010693	39434	QALLXIUHRUVULB-OKFWSBNLSA-N	CC(=O)O[C@@H](COP([O-])(=O)OCC[N+](C)(C)C)COC(=O)CCCCCCC/C=C\CCCCCC
**535,3645**	8,235748	PC(O-19:1)/LysoPE(22:1)/PE(22:1)/PC(19:1)	C27H54NO7P	3		LMGP01020070	40097	FZTHXYOLVRRAMJ-AREMUKBSSA-N	CCCCCCCCCCCCCCCCOC[C@@H](COP([O-])(=O)OCC[N+](C)(C)C)OC(=O)C=C
**535,3998**	9,048944	PC(O-20:0)/11alpha-(4-dimethylaminophenyl)-1alpha,25-dihydroxyvitamin D3 / 11alpha-(4-dimethylaminophenyl)-1alpha,25-dihydroxycholecalciferol/dl-	C28H58NO6P	3			40228		

		alpha-Tocopherol nicotinate;							
**537,3427**	6,621509	PC(18:0)/PS(P-20:0)	C26H52NO8P	3		LMGP01010989	40025	KDXVSDQFHRZPKV-RUZDIDTESA-N	CC(=O)OC[C@@H](COP([O-])(=O)OCC[N+](C)(C)C)OC(=O)CCCCCCCCCCCCCCC
**537,3788**	8,903561	LysoPE(22:0)	C27H56NO7P	3	HMDB0011490	LMGP02050054	62278	NVLXNEISHNIEBO-AREMUKBSSA-N	CCCCCCCCCCCCCCCCCCCCCC(=O)O[C@@H](COP(O)(=O)OCCN)CO
**537,3796**	9,109756	PC(O-19:0)/LysoPE(22:0)/PE(22:0)	C27H56NO7P	3		LMGP01020068	40095	IMFQQPWSHMFNRT-AREMUKBSSA-N	CCC(=O)O[C@@H](COCCCCCCCCCCCCCCCCCC)COP([O-])(=O)OCC[N+](C)(C)C
**537,4147**	10,150806	PC(O-20:0)	C28H60NO6P	3			40181		
**539,3226**	7,16983	LPS(19:0)	C25H50NO9P	3		LMGP03050028	78855	QZTQWDNDCFZUCX-PKTZIBPZSA-N	CCCCCCCCCCCCCCCCCCC(=O)OC[C@@H](O)COP(O)(=O)OC[C@H](N)C(O)=O
**541,3168**	7,0437965	PC(20:5)	C28H48NO7P	3	HMDB0010397	LMGP01050050	40316	PDIGSOAOQOXRDU-WJPZTBRDSA-N	CC/C=C\C/C=C\C/C=C\C/C=C\C/C=C\CCCC(=O)OC[C@@H](O)COP([O-])(=O)OCC[N+](C)(C)C
**541,3396**	7,5290046	dihydroxycholestanoyl taurine	C29H51NO6S	3		LMST05040008	57994	JPTLXYYSWLLUOV-NZSCTMKGSA-N	C[C@@]12CCC3C(C1CCC2[C@@H](C)CCC[C@H](C)C(=O)NCCS(O)(=O)=O)[C@@H](O)C[C@H]1C[C@@H](O)CC[C@]13C
**541,3404**	7,667154	dihydroxycholestanoyl taurine	C29H51NO6S	3		LMST05040008	57994	JPTLXYYSWLLUOV-NZSCTMKGSA-N	C[C@@]12CCC3C(C1CCC2[C@@H](C)CCC[C@H](C)C(=O)NCCS(O)(=O)=O)[C@@H](O)C[C@H]1C[C@@H](O)CC[C@]13C
**543,3321**	7,177292	PC(20:4)/LysoPC(20:4)	C28H50NO7P	3		LMGP01050121	46690	IGJKYDBBINVMLH-JXRLJXCWSA-N	CCCCC/C=C\C/C=C\C/C=C\C/C=C\CCCC(=O)O[C@@H](CO)COP([O-])(=O)OCC[N+](C)(C)C
**543,3325**	7,348899	PC(20:4)/LysoPC(20:4)	C28H50NO7P	3			40315		
**545,3479**	7,495645	LysoPC(20:3)	C28H52NO7P	3	HMDB0010394	LMGP01050133	61704	BBNHCUBQEQJHIG-FZZJNMCHSA-N	CCCCC/C=C\C/C=C\C/C=C\CCCCCCC(=O)OC[C@@H](O)COP([O-])(=O)OCC[N+](C)(C)C
**545,3481**	7,6089	LysoPC(20:3)	C28H52NO7P	3	HMDB0010394	LMGP01050133	61704	BBNHCUBQEQJHIG-FZZJNMCHSA-N	CCCCC/C=C\C/C=C\C/C=C\CCCCCCC(=O)OC[C@@H](O)COP([O-])(=O)OCC[N+](C)(C)C
**547,3633**	7,902995	PC(O-20:2)	C28H54NO7P	3		LMGP01020158	40019	ICPPVEDRGDADTQ-IZZMTCJOSA-N	CCCCC/C=C\C/C=C\CCCCCCCCCOC[C@@H](COP([O-])(=O)OCC[N+](C)(C)C)OC(C)=O
**547,3633**	8,040506	LysoPC(O-20:2)	C28H54NO7P	3		LMGP01020158	40019	ICPPVEDRGDADTQ-IZZMTCJOSA-N	CCCCC/C=C\C/C=C\CCCCCCCCCOC[C@@H](COP([O-])(=O)OCC[N+](C)(C)C)OC(C)=O
**549,3774**	8,516408	Pyrrolidino PAF C-16/PC(P-20:0)/PC(20:1)/PC(O-20:1)	C28H56NO7P	3			62930		
**549,3791**	8,702327	PC(20:1)/PC(P-20:0)/Pyrrolidino PAF C-16	C28H56NO7P	3		LMGP01050047	40313	GJTDRNFWIDPARY-GTPZACKGSA-N	CCCCCCCCCC/C=C\CCCCCCCC(=O)OC[C@@H](O)COP([O-])(=O)OCC[N+](C)(C)C
**551,322**	6,1284285	PS(20:1/0:0)	C26H50NO9P	3		LMGP03050020	78847	WQWSUENXYNSINA-KCVNTPOGSA-N	CCCCCCCC/C=C\CCCCCCCCCC(=O)OC[C@@H](O)COP(O)(=O)OC[C@H](N)C(O)=O
**551,322**	6,5090704	PS(20:1)	C26H50NO9P	3		LMGP03050020	78847	WQWSUENXYNSINA-KCVNTPOGSA-N	CCCCCCCC/C=C\CCCCCCCCCC(=O)OC[C@@H](O)COP(O)(=O)OC[C@H](N)C(O)=O
**551,3934**	8,822981	PC(20:0)/PAF C-18	C28H58NO7P	3			40164		
**551,3949**	9,710144	LysoPC(20:0)	C28H58NO7P	3	HMDB0010390	LMGP01050045	40311	UATOAILWGVYRQS-HHHXNRCGSA-N	CCCCCCCCCCCCCCCCCCCC(=O)OC[C@@H](O)COP([O-])(=O)OCC[N+](C)(C)C

**557,3102**	6,192817	PC(20:4)	C28H48NO8P	3			39147		
**562,4201**	10,640892	Campesteryl glucoside	C34H58O6	3		LMST01031126	103408	FWNZEKQVBDXWKA-ZCWREFTMSA-N	CC(C)[C@@H](C)CC[C@H](C)[C@@H]1CC[C@@H]2[C@H]3CC=C4C[C@@H](CC[C@@]4(C)[C@@H]3CC[C@]21C)O[C@H]1OC(CO)[C@H](O)C(O)C1O
**567,3173**	6,245624	PS(10:0/10:0)/Glucuronosylhyodeoxycholate	C26H50NO10P	3		LMGP03010022	40807	LRIPXDCMGANCAE-PKTZIBPZSA-N	CCCCCCCCCC(=O)OC[C@@H](COP(O)(=O)OC[C@@H](N)C(O)=O)OC(=O)CCCCCCCCC
**567,3322**	7,3212414	PC(22:6)	C30H50NO7P	3			40321		
**567,3542**	7,7095814	PS(21:0)	C27H54NO9P	3		LMGP03050026	78853	IFZCAKDVWKLKEK-RPBOFIJWSA-N	CCCCCCCCCCCCCCCCCCCCC(=O)OC[C@@H](O)COP(O)(=O)OC[C@H](N)C(O)=O
**567,3554**	7,8525476	PS(21:0)	C27H54NO9P	3		LMGP03050026	78853	IFZCAKDVWKLKEK-RPBOFIJWSA-N	CCCCCCCCCCCCCCCCCCCCC(=O)OC[C@@H](O)COP(O)(=O)OC[C@H](N)C(O)=O
**569,346**	7,4238377	LysoPC(22:5)	C30H52NO7P	3	HMDB0010403	LMGP01050143	61712	BIEOSECQPGGZMF-NAIWXXGBSA-N	CC/C=CC/C=CC/C=CC/C=CC/C=CCCCCCC(=O)OC[C@@H](O)COP([O-])(=O)OCC[N+](C)(C)C
**569,3475**	7,680326	LysoPC(22:5)	C30H52NO7P	3	HMDB0010403	LMGP01050143	61712	BIEOSECQPGGZMF-NAIWXXGBSA-N	CC/C=CC/C=CC/C=CC/C=CC/C=CCCCCCC(=O)OC[C@@H](O)COP([O-])(=O)OCC[N+](C)(C)C
**571,3617**	7,899891	PC(22:4)/Diprenylpaxilline	C30H54NO7P	3	HMDB0010401	LMGP01050124	61710	ZOJBSSVHFSBHMP-JJJSWPRASA-N	CCCCC/C=C\C/C=C\C/C=C\C/C=C\CCCCCC(=O)OC[C@@H](O)COP([O-])(=O)OCC[N+](C)(C)C
**577,3736**	8,338145	PC(42:1)	C29H56NO8P	3		LMGP01010673	39414	SOZUKXUDKQAUFE-HHHXNRCGSA-N	CCCCCCCCCCCCCCCC(=O)OC[C@@H](COP([O-])(=O)OCC[N+](C)(C)C)OC(=O)CCC=C
**579,3528**	8,013977	PC(20:0(CHO))/1-palmitoyl-4-oxobutanoyl-sn-glycero-3-phosphocholine/OB-PC/PS(22:1)	C44H81NO8P	3					
**579,389**	8,415118	PC(21:0)/PE(24:0)	C29H58NO8P	3		LMGP01010829	39570	BTUFMQGOQOCKKM-HHHXNRCGSA-N	CCC(=O)O[C@@H](COP([O-])(=O)OCC[N+](C)(C)C)COC(=O)CCCCCCCCCCCCCCCCC
**593,3689**	8,116122	PC(16:0/5:0(CHO))/palmitoyl(oxovaleroyl)glycerophosphocholine/POV-PC/OV-PC	C29H56NO9P	3					
**605,4047**	8,590739	PE(26:0)	C31H60NO8P	3		LMGP02010359	76594	YKTVMHWVXDZXEQ-HQGHLRICSA-N	CCCCCCCCCCCC(=O)OC[C@@H](COP(O)(=O)OCCN)OC(=O)CCCCCCC/C=C\CCCC
**609,3643**	7,812722	LysoPC(21:0)	C28H54NO10P	3					
**618,2778**	8,923297	PI(20:5)	C29H47O12P	3		LMGP06050026	81191	NAVULVNLGJVPQU-DFBNLFNOSA-N	CC/C=C\C/C=C\C/C=C\C/C=C\C/C=C\CCCC(=O)OC[C@@H](O)COP(O)(=O)O[C@@H]1C(O)[C@H](O)C(O)C(O)C1O
**620,2958**	8,775469	PI(20:4)/arachidonoylglycerophosphomyoinositol	C29H49O12P	3		LMGP06050006	46748	LXUGKKVCSTYZFK-MUOQEERCSA-N	CCCCC/C=C\C/C=C\C/C=C\C/C=C\CCCC(=O)OC[C@@H](O)COP(O)(=O)O[C@@H]1C(O)[C@H](O)C(O)C(O)C1O
**649,3969**	7,903001	PC(17:1(CHO-OH))/PS(26:1)/palmitoyl(hydroxyoxooctenoyl)glycerophо	C32H60NO10P	3					

		sphocholineHOOA-PC								
**649,4316**	8,972283	PC(25:0 CHO)	C33H64NO9P	3			LMGP20010008	82381	PPTNNIINSOQWCE-WJOKGBTCSA-N	CCCCCCCCCCCCCCCC(=O)OC[C@@H](COP([O-])(=O)OCC[N+](C)(C)C)OC(=O)CCCCCCCC=O
**649,432**	9,514757	PC(16:0/9:0)/palmitoyloxononanoyl)-sn-glycerophosphocholine	C33H64NO9P	3						
**663,4475**	9,803808	PC(26:0(COH))/palmitoyl(oxodecanoyl)-sn-glycero-3-phosphocholine/OD-PC/PS(P-28:0)	C34H66NO9P	3						
**665,427**	8,336828	PC(25:0(COOH))/palmitoylazelaoylglycerophosphocholine/AZ-PC/PS(27:0)/PAz-PC	C33H64NO10P	3						
**677,4635**	10,12566	PC(16:0/11:0)/palmitoyl(oxoundecanoyl)-sn-glycerophosphocholine/OU-PC/PS(P-29:0)	C35H68NO9P	3						
**689,4282**	9,229844	PS(29:2)	C35H64NO10P	3			LMGP03010278	77942	YZWDXSZQZCNQND-JKHFDAINSA-N	CCCC/C=C\C/C=C\CCCCCCCC(=O)OC[C@@H](COP(O)(=O)OC[C@@H](N)C(O)=O)OC(=O)CCCCCCCCCCC
**691,4412**	8,511854	PS(29:1)	C35H66NO10P	3			LMGP03010248	77912	ODAOIMAACFDKOX-UKODLXMQSA-N	CCCCCCC/C=C\CCCCCCCC(=O)OC[C@@H](COP(O)(=O)OC[C@@H](N)C(O)=O)OC(=O)CCCCCCCCCCC
**691,4433**	8,947102	PS(29:1)	C35H66NO10P	3			LMGP03010051	77715	AZRMVTACVQNFOG-UKODLXMQSA-N	CCCCCCCCCCCC(=O)OC[C@@H](COP(O)(=O)OC[C@@H](N)C(O)=O)OC(=O)CCCCCCC/C=C\CCCCCCC
**693,4582**	9,350933	PS(29:0)	C35H68NO10P	3		HMDB0112273	LMGP03010930	78594	RDGOWVQONPEGML-ZWXJPIIXSA-N	CCCCCCCCCCCCCC(=O)OC[C@@H](COP(O)(=O)OC[C@@H](N)C(O)=O)OC(=O)CCCCCCCCCCCCCC
**714,4812**	5,7083187	Deoxymyxol fucoside	C46H66O6	3	C15940			64118	LQRQZOJVMFJJLX-LJSSMQLHSA-N	CC1=C(/C=C/C(C)=C/C=C/C(C)=C/C=C/C=C(C)/C=C/C=C(C)/C=C/C=C(C)/C=C/[C@H](O[C@@H]2O[C@@H](C)[C@@H](O)[C@@H](O)[C@@H]2O)C(C)(C)O)C(C)(C)CCC1
**716,4579**	5,698094	PG(32:3)	C38H69O10P	3			LMGP04010368	79193	PZHKWBXQKOJTIF-GIJSESEMSA-N	CCCCCCCCCCCCCC(=O)O[C@@H](COP(O)(=O)OC[C@@H](O)CO)COC(=O)CCCC/C=C\C/C=C\C/C=C\CCCCC
**732,4243**	5,704729	Kurilensoside J	C37H64O14	3			LMST05050020	84940	KHUZIKFEVDFTLS-ISWDZEJHSA-N	CC(C)[C@@H](CC[C@H](C)[C@@H]1C[C@@H](O)[C@@H]2[C@@]3(O)C[C@H](O)[C@@H]4[C@H](O)[C@@H](CC[C@@]4(C)[C@@H]3CC[C@@]12C)O[C@H]1OC[C@H](O)C(O)C1OC)O[C@H]1O[C@H](O)C(O)C1O
**749,3086**	8,553471	Debenzoyltaxol/Phenylisoserinoyl baccatin III	C40H47NO13	3	C16524			64037	DLACRDONFBNRSP-UHTFTNQHSA-N	CC(=O)O[C@H]1C(=O)[C@@]2(C)C([C@H](OC(=O)c3ccccc3)[C@]3(O)C[C@H](OC(=O)[C@H](O)[C@@H](N)c4ccccc4)C(C)=C1C3(C)C)[C@]1(OC(C)=O)CO[C@@H]1C[C@@H]2O
**755,5472**	11,690027	PC(34:3)	C42H78NO8P	3		HMDB0008199	LMGP01011677	59645	RAPKGPDUGWBPHR-QWFQJEORSA-N	CCCCCCCCCCCCCCCC(=O)O[C@@H](COP([O-])(=O)OCC[N+](C)(C)C)COC(=O)CCCCCCC/C=C\C/C=

										C\C/C=C\CC
**792,4981**	5,709619	PG(38:7)	C44H73O10P	3			LMGP04010660	79485	YEAIPFFGULDBAZ-SEVDQDBBSA-N	CCCCC/C=C\C/C=C\CCCCCCCC(=O)O[C@@H](COP(O)(=O)OC[C@@H](O)CO)COC(=O)CCC/C=C\C/C=C\C/C=C\C/C=C\C/C=C\CC
**922,6826**	5,5540676	PI(O-41:0)	C50H99O12P	3			LMGP06020066	81050	DAJWPQITDOHFGF-ULZUSFMRSA-N	CCCCCCCCCCCCCCCCCCCCOC[C@@H](COP(O)(=O)O[C@H]1C(O)[C@@H](O)C(O)C(O)C1O)OC(=O)CCCCCCCCCCCCCCCCCCC
**942,6242**	5,759816	PI(42:4)	C51H91O13P	3			LMGP06010688	80710	OILYISDPYNFGDU-JSQJEHOFSA-N	CCCCCCCCCCCCCCCCCCCCCC(=O)OC[C@@H](COP(O)(=O)O[C@H]1C(O)[C@@H](O)C(O)C(O)C1O)OC(=O)CCC/C=C\C/C=C\C/C=C\C/C=C\CCCCC
**950,6785**	5,7493453	PI(42:0)/PC(DiMe(13,5)/DiMe(11,5))/PC(MonoMe(13,5)/MonoMe(13,5))/PC(DiMe(11,5)/DiMe(13,5))	C51H99O13P	3			LMGP06010685	80707	AMGJMFGIJRCTHV-OFJFGGNTSA-N	CCCCCCCCCCCCCCCCCCCCCC(=O)OC[C@@H](COP(O)(=O)O[C@H]1C(O)[C@@H](O)C(O)C(O)C1O)OC(=O)CCCCCCCCCCCCCCCCCCC
**1059,5627**	5,40907	Bradykinin	C50H73N15O11	3	C00306	HMDB0004246		58231	QXZGBUJJYSLZLT-FDISYFBBSA-N	N[C@@H](CCCNC(N)=N)C(=O)N1CCC[C@H]1C(=O)N1CCC[C@H]1C(=O)NCC(=O)N[C@@H](CC1=CC=CC=C1)C(=O)N[C@@H](CO)C(=O)N1CCC[C@H]1C(=O)N[C@@H](CC1=CC=CC=C1)C(=O)N[C@@H](CCCNC(N)=N)C(O)=O
**2161,2676**	7,658147	(Mannosyl)3-(N-acetylglucosaminyl)2-diphosphodolichol	C114H190N2O32P2	3		HMDB0012119		62794	BKUXVZCTYBVOAV-JLQUOVFMSA-N	OC[C@H]1O[C@H](OC[C@H]2O[C@@H](O[C@H]3[C@H](O)[C@@H](NC(C)=O)[C@@H](O[C@@H]3CO)O[C@H]3[C@H](O)[C@@H](NC(C)=O)[C@@H](O[C@@H]3CO)OP(=O)(O)OP(=O)(O)OCCC(C)CC\C=C(/C)CC\C=C(/C)CC\C=C(/C)CC\C=C(/C)CC\C=C(/C)CC\C=C(/C)CC\C=C(/C)CC\C=C(/C)CC\C=C(/C)CC\C=C(/C)CC\C=C(/C)CC\C=C(/C)CC\C=C(/C)CC\C=C(/C)CCC=C(C)C)[C@@H](O)[C@@H](O[C@H]3O[C@H](CO)[C@@H](O)[C@H](O)[C@@H]3O)[C@@H]2O)[C@@H](O)[C@@H](O)[C@@H]1O
**2174,0803**	5,981447	NeuAcalpha2-8NeuAcalpha2-3Galbeta1-4GlcNAcbeta1-3Galbeta1-4GlcNAcbeta1-3Galbeta1-4Glcbeta-Cer(d18:1/16:0)	C96H167N5O49	3			LMSP0601GB01	57382	BEOTWPTXPHLJBM-ZDBWLZBKSA-N	
